# Inhibition of choline metabolism in an angioimmunoblastic T-cell lymphoma preclinical model reveals a new metabolic vulnerability as possible target for treatment

**DOI:** 10.1186/s13046-024-02952-w

**Published:** 2024-02-06

**Authors:** Adrien Krug, Marie Tosolini, Blandine Madji Hounoum, Jean-Jacques Fournié, Roger Geiger, Matteo Pecoraro, Patrick Emond, Philippe Gaulard, François Lemonnier, Jean-Ehrland Ricci, Els Verhoeyen

**Affiliations:** 1 Université Côte d’Azur, INSERM, C3M, 06204 Nice, France; 2 Equipe Labellisée Ligue Contre Le Cancer, 06204 Nice, France; 3 Centre de Recherches en Cancérologie de Toulouse, CRCT, Université de Toulouse, CNRS, Université Toulouse III-Paul Sabatier, Inserm, Toulouse, France; 4 Institute for Research in Biomedicine (IRB), Università della Svizzera italiana, Bellinzona, Switzerland; 5 Institute of Oncology Research (IOR), Università della Svizzera Italiana, Bellinzona, Switzerland; 6 Labex TOUCAN, Toulouse, France; 7 UMR iBrain, Université de Tours, Inserm, Tours, France; 8 Université Paris-Est Créteil, Institut Mondor de Recherche Biomedicale, Creteil, INSERMU955 France; 9 AP-HP, Groupe Hospitalo-Universitaire Chenevier Mondor, Département de Pathologie, 94010 Créteil, France; 10 AP-HP, Groupe Hospitalo-Universitaire Chenevier Mondor, Service Unité Hémopathies Lymphoides, 94010 Créteil, France; 11 CIRI, Université de Lyon, INSERM U1111, ENS de Lyon, University Lyon1, CNRS, UMR5308, Lyon, 69007 France

**Keywords:** Choline, AITL, Lipid metabolism, CDP-choline pathway, CDP-ethanolamine pathway, Choline kinase, Cancer therapy, T-cell lymphoma

## Abstract

**Background:**

Angioimmunoblastic T-cell lymphoma (AITL) is a malignancy with very poor survival outcome, in urgent need of more specific therapeutic strategies. The drivers of malignancy in this disease are CD4^+^ follicular helper T cells (Tfh). The metabolism of these malignant Tfh cells was not yet elucidated. Therefore, we decided to identify their metabolic requirements with the objective to propose a novel therapeutic option.

**Methods:**

To reveal the prominent metabolic pathways used by the AITL lymphoma cells, we relied on metabolomic and proteomic analysis of murine AITL (mAITL) T cells isolated from our established mAITL model. We confirmed these results using AITL patient and healthy T cell expression data.

**Results:**

Strikingly, the mAITL Tfh cells were highly dependent on the second branch of the Kennedy pathway, the choline lipid pathway, responsible for the production of the major membrane constituent phosphatidylcholine. Moreover, gene expression data from Tfh cells isolated from AITL patient tumors, confirmed the upregulation of the choline lipid pathway. Several enzymes involved in this pathway such as choline kinase, catalyzing the first step in the phosphatidylcholine pathway, are upregulated in multiple tumors other than AITL. Here we showed that treatment of our mAITL preclinical mouse model with a fatty acid oxydation inhibitor, significantly increased their survival and even reverted the exhausted CD8 T cells in the tumor into potent cytotoxic anti-tumor cells. Specific inhibition of Chokα confirmed the importance of the phosphatidylcholine production pathway in neoplastic CD4 + T cells, nearly eradicating mAITL Tfh cells from the tumors. Finally, the same inhibitor induced in human AITL lymphoma biopsies cell death of the majority of the hAITL PD-1^high^ neoplastic cells.

**Conclusion:**

Our results suggest that interfering with choline metabolism in AITL reveals a specific metabolic vulnerability and might represent a new therapeutic strategy for these patients.

**Supplementary Information:**

The online version contains supplementary material available at 10.1186/s13046-024-02952-w.

## Background

Peripheral T-cell lymphomas (PTCLs) represent 12–15% of all lymphoid malignancies in Western countries and include over 20 entities [1]. However, chemotherapy regimens that cure many patients with B cell lymphomas have produced very disappointing results in PTCL. One of the most prominent PTCLs is angioimmunoblastic T-cell lymphoma (AITL), which is a devastating disease, affecting mostly elderly patients, characterized by general lymphadenopathy, splenomegaly and hepatomegaly [2, 3]. AITL is recognized as a CD4 T-cell disorder derived from the malignant transformation of T follicular helper (Tfh) cells, associated with germinal center (GC) B cell dysregulation [2]. AITL disease outcome is poor, with an overall 5-year survival rate of 30% and patients usually do not respond to cytotoxic chemotherapeutic treatment [4, 5]. Though several new therapeutic agents such as epigenetic modifiers have been proposed [6], of which some were tested in the clinic, little progress has been made in AITL therapies. Thus, optimal management of AITL and PTCL represents an unmet medical need. To address this medical challenge, we need to acquire more knowledge about the biology of AITL, which is challenging due to the rareness of these cancers. Therefore, a preclinical mouse model of AITL is invaluable for testing of new therapeutic options for AITL disease. Previously, we generated a unique in vivo model for AITL by overexpressing GAPDH, one of the key glycolytic enzymes recently emerging as a key player in T-cell survival, development and function of the T-cell compartment [7, 8]. These mice developed a T malignancy which mimicked human AITL very closely including its appearance at older age, hepatomegaly, splenomegaly, lymph node enlargement, strong inflammation, skin rash and ascite accumulation. By transcriptional profiling, genetic approaches and immuno-phenotyping of the plck-GAPDH tumors, we demonstrated that this mouse model recapitulated multiple features of the human AITL disease [9, 10]. The mice that developed the murine lymphoma were characterized by an abundant tumor micro-environment including for the majority germinal center (GC) B cells and neoplastic T cells. These have a T follicular helper (Tfh) phenotype (CD4^+^ PD1^high^, CXCR5^+^, ICOS^+^) equivalent to human AITL [9, 11]. Using gene set enrichment analysis, we identified in both murine and human AITL Tfh cells an upregulation of the non-canonical NF-κB pathway. Therapeutic intervention with NF-κB Induced Kinase (NIK) inhibitors proved to be an effective treatment for mAITL in vivo, which was confirmed in AITL patient biopsies in vitro [9]. This underlined the validity of the unique mAITL lymphoma model to address the challenges posed by the low prevalence of human AITL, which closely resembles this murine model.

Up to now, the metabolic adaptation of the malignant Tfh cells known to be the drivers in AITL disease remains to be identified. Therefore, we wanted to reveal the metabolic addiction of the neoplastic Tfh cells in AITL in order to propose novel therapeutic options. Integrating proteomic and metabolomic data obtained from mAITL lymphoma CD4 + PD1^high^ T cells and wild-type (WT) CD4 + cells, allowed us to identify a specific lipid metabolic pathway involved. We identified the choline pathway as strongly active in the mAITL cells. Gene expression data from purified CD4 + Tfh cell from human tumors confirmed the upregulation of this part of the Kennedy Pathway also in human AITL. Therapeutic intervention by relevant candidate drugs inhibiting fatty acid oxidation and a Chokα inhibitor were tested in our mAITL preclinical model. It resulted in increased mice survival through elimination of the CD4 + PD1^high^ Tfh cells and associated GC B cells and interestingly by reactivation of cytotoxic CD8 T cells in the tumor microenvironment. Finally, we confirmed that inhibiting choline lipid metabolism in human AITL biopsies selectively eliminated the neoplastic Tfh cells in agreement with data obtained in our preclinical mAITL model. This revealed a new metabolic vulnerability in AITL cancer as a possible therapeutic target.

## Methods

### Plck-GAPDH mouse model

Plck-GAPDH mice were generated in our lab and described in our previous study [9].

### Tumor transplantation into NOD/SCIDγc-/- (NSG) mice

Detailed description for tumor engraftement in NSG (RRID:IMSR_JAX:005557) is given in supplementary methods.

All experimental procedures were carried out in compliance with protocols approved by the local ethical and experimentation committee (SBEA, Nice, France, autorisation N° 28,790–2020121715244498 and B0608820).

### Drug administration in plck-GAPDH lymphoma engrafted NSG mice

#### Etomoxir treatment

Splenic lymphoma cells from plck-GAPDH mice were injected intravenously into recipient NSG mice. Fourteen days after tumor cell injection, lymphoma engrafted NSG mice were treated by IP injection with etomoxir (20 mg/kg, Sigma-Aldrich), an inhibitor of fatty acid oxidation or with vehicle (100 μl PBS/mouse). The treatment regimen consisted in 3 injections per week.

### MN58b treatment

Fourteen days after lymphoma engraftment, NSG mice were treated with MN58b by IP injection (4 mg/kg) or with vehicle (10% DMSO + 90% NaCl 0,9%). The treatment regimen consisted in 2 injections/week as indicated in Fig. 6D.

For all NSG recipient mice were sacrificed at humane endpoint (> 10% weight loss or palpable splenomegaly) or before. Single cell suspensions were prepared from the spleen, LN and liver for immunophenotypic analysis by FACS.

### Isolation of primary mouse T cells

Described in supplementary material and methods.

### Proteomics and metabolite analysis

Detailed description can be found in supplementary methods.

### Flow cytometry and antibodies for murine immune cells

Antibodies used for detailed phenotyping or intracellular staining by flow cytometry of murine T, B are listed here and acquired from Miltenyi: CD3 APCcy7 (130–102-306, RRID:AB_2660402), CD4 FITC (130–102-54, RRID:AB_2659902); CD8 PEcy7 (130–119-123, RRID:AB_2733250), B220 FITC (130–110-845, RRID:AB_2658273), PD-1 PE (130–111-800, RRID:AB_2656934), CXCR5 APC (130–103-113, RRID:AB_2655792), ICOS-VB (130–100-639, RRID:AB_2656917) or BD Pharmingen/ CD19 PE (553,786, RRID:AB_395050), CD95 VB (562,633, RRID:AB_2737690), INFγ APC (554,413, RRID:AB_398551), GL-7 APC (561,529, RRID:AB_10716056) or E-bioscience: Perforin PE (12–9392-82, RRID:AB_466243), Granzyme B PEcy7 (25–8898-82, RRID:AB_10853339).

Staining with MitoTracker® Green (Fisher Scientific, M7514; 150 nM) was performed according to manufacturer’s instructions followed by surface staining before FACS analysis.

For analysis of ROS content by FACS the CellROX Green flow cytometry Assay kit (Thermofisher, C10444) was used according to manufacurer’s instructions.

For intracellular staining of Granzyme B, Perforin and IFNγ splenocytes were stimulated for 5 h in PMA (phorbol 12-myristate-13-acetate, Sigma, # P8139)/ionomycin (Sigma, # I0634) in the presence of Golgi-stop (BD Biosciences Cat# 555,029, RRID:AB_2869014) and upon surface staining (anti-CD4 and anti-CD8) cells were fixed and permeabilized using the Cytofix/Cytoperm kit and protocol (BD Biosciences Cat# 554,714, RRID:AB_2869008).

All stainings were detected using a Miltenyi Bioscience MACSQuant Analyzer 10 (RRID:SCR_020268). Analysis of the FACS data was performed using MACSquantify Version 2.11 (Miltenyi, RRID:SCR_020943) and FlowJo Software (RRID:SCR_008520).

### Ex vivo treatment of mAITL tumor cells and WT splenocytes

The mAITL tumor cells or WT splenocytes were cultured at 5E5 cells/well in RPMI media supplemented with 10% FCS, 50 nM β-Mercaptoethanol and following murine cytokines obtained from Peprotech: 25 ng mL^−1^ IL-6, 50 ng mL^−1^ IL-21, 10 ng mL^−1^ IL-7, 10 ng mL^−1^ IL-15 and 5 ng mL^−1^ IL-2. MN58b (5 μM) was added to the medium for 72 h as indicated. Etomoxir was added at the indicated doses to the cells in the medium described above in the absence or presence of N-Acetyl-L-cysteine (NAC, 2 mM) for 96 h. Ranolazine was added at the indicated doses to the tumor cells and medium described above for 96 h. DAPI staining was performed to evaluate cell death by FACS after surface-staining for CD4/CD8/PD1/CD19 using antibodies described above.

### AITL patient and healthy donor Tfh cell gene expression data

Healthy Tfh cells were isolated from healthy donors who got their tonsils removed. The tonsils were perfused with RPMI medium. Subsequently, T cells were isolated using the Pan T-cell kit (Miltenyi) and then further purified by FACS sorting for the CD4 + CXCR5 + ICOS + PD-1 + cells. Then cells were pelleted and processed for RNA extraction. For AITL Tfh cells were isolated from enlarged lymph nodes from patients using the same isolation steps as healthy donor Tfh cells. RNA was extracted from purified AITL Tfh cells and Tfh cells from healthy donors. Libraries were prepared and sequenced as described [9]. Affymetrix data are available (GSE232609, confidential Token for access: gnsjgkyozjulnmn) and were quantified by RSEM software 1.2.25 [12] using GRCh38v97 reference genome. These data were compared to public raw data available for healthy donor Tfh, naïve, memory, regulatory and stem cell memory CD4 + T cells downloaded from GEO datasets GSE61697, GSE65010, GSE66384 and GSE71566. Raw files were downloaded and normalized together using RMA methods. GEO number for the Tfh AITL dataset and healthy donor Tfh CD4 data set: GSE19069, GSE58445 et E-TABM-783 (https://www.ebi.ac.uk/biostudies/arrayexpress /studies/E-TABM-783).

For analysis, expression data were normalized with z-score methods when specified and illustrated with heatmaps using R software (3.3.2). Statistical differences were verified using an unpaired two-tailed Wilcoxon signed rank test versus the specified controls. Metabolic pathway analysis was performed using KEGG data base: https:// www.ncbi.nlm.nih.gov/pubmed/10592173. For other pathway analysis we used the Reactome data base (https://reactome.org/) [13] and MsigDB (https://www.gsea-msigdb.org/gsea/msigdb/) [14].

### AITL biopsies and healthy donor PBMCs

Adult healthy blood samples were collected in sterile tubes containing the anti-coagulant, citrate-dextrose (ACD, Sigma, France). Samples of AITL patients were retrospectively obtained from the onco-hematology laboratory of the ‘Necker-Enfants Malades’ hospital in Paris (France). The cells were suspended after mechanical dissection of the lymph node biopsy and frozen in 10% DMSO in FCS. All human blood and tissues were obtained after informed consent and approval was obtained by the ethical commission of the hospitals according to the Helsinki declaration. Human T cells were isolated from peripheral adult blood isolated by negative selection using a Pan T cell enrichment Kit (Miltenyi, #130–096-535) according to manufacturer’s instructions.

### Ex vivo treatment of AITL patient cells

The human AITL tumor cells or healthy donor PBMCs were cultured at 5E5 cells/well in RPMI media supplemented with 10% FCS, and following human cytokines obtained from Peprotech: 25 ng mL^−1^ IL-6, 50 ng mL^−1^ IL-21, 10 ng mL^−1^ IL-7, 10 ng mL^−1^ IL-15 and 5 ng mL^−1^ IL-2. MN58b (5 μM) was added to the medium for 72 h as indicated. DAPI staining was performed to evaluate cell death by FACS after surface-staining for CD4/CD8/PD1/CD19 with following antibodies: anti-hCD3 APC-Cy7 (#130–113-136, RRID:AB_2725964), anti-hCD4 APC (#130–113-222, RRID:AB_2726033), anti-hCD8 PE Cy7 (#130–110-680, RRID:AB_2659245), anti-hPD1 PE (#130–120-382, RRID:AB_2752069), anti-hCD19 VB (#130–120-031, RRID:AB_2784030), anti-hCD19 FITC (#130–113-645, RRID:AB_2726198).

### Quantification and statistical analysis

Statistical analysis was conducted using Microsoft excel 2013 and Prism software v6.0 (GraphPad Software, La Jolla, CA, USA). Results are indicated as means ± SD (standard deviation) in the figure legends unless indicated otherwise. For statistical testing of significance a student’s t-test or ONE way ANOVA was used followed by Tukey range test to assess the significance among pairs of conditions; p-values are indicated in the figure legends. A *p*-value < 0.05 was considered to indicate statistical significance. Mice survival curves were evaluated using Log-rank test to determine significance. All flow cytometry data shown are representative of at least 3 reproduced experiments. Gene set enrichment analysis was performed as described above.

## Results

### Lipid metabolism shows significant changes in murine and human AITL Tfh cells compared to their healthy counterpart CD4 + T-cell subsets

**Fig. 1:**
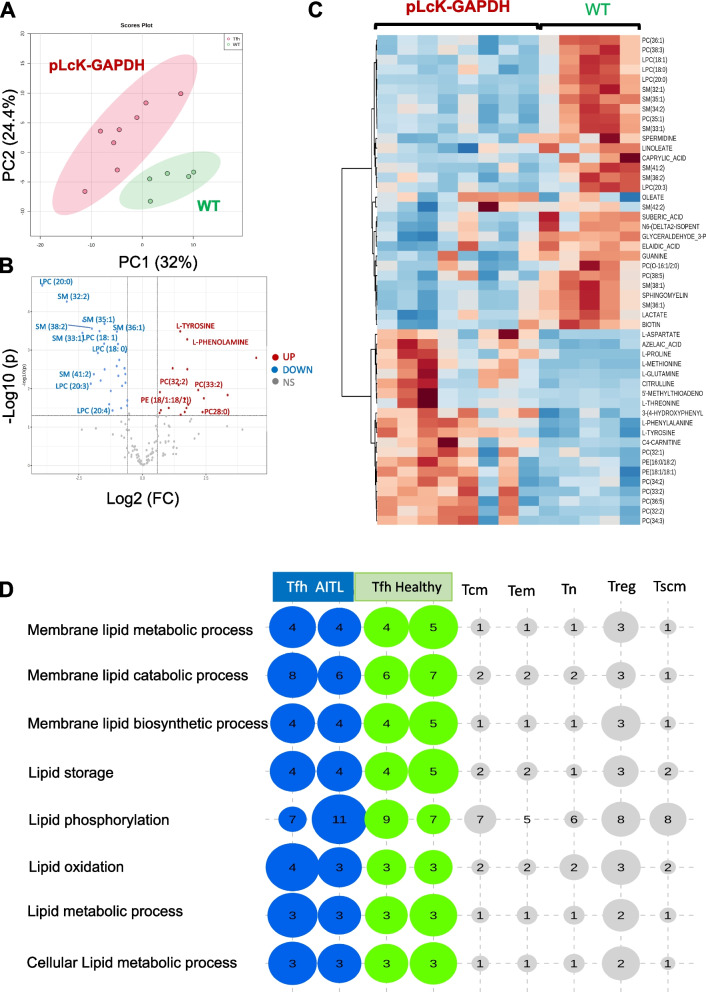
Significant differences in lipid metabolites are detected in murine and human AITL Tfh cells compared to healthy T cells. CD4 + PD-1^high^ cells were isolated from pLck-GAPDH mouse lymphoma and compared to WT CD4 + splenocytes for metabolite analysis. Principle component analysis for the two groups in is shown in (**A**) and data are represented in the volcanoplot (**B**). **C** Heatmap of the 50 metabolites showing more than 1.5 fold log2 change between the mAITL CD4 + PD-1^high^ T cells and splenic CD4 + T cells. **D** Lipid metabolism pathway analysis for the GSEA data from AITL patient (*n* = 8), healthy Tfh cells (*n* = 14), central memory T cells (Tcm) (*n* = 6), effector memory T cells (Tem) (*n* = 6), naive T cells (Tn) (*n* = 6), regulatory T cells (T reg) (*n* = 13) and stem cell memory T cell (Tscm) (*n* = 6). Bubble representation (Bubbles size and numbers represents the sample enrichment score (SES); *p*-values are indicated in supplementary Fig. 1A)

Angioimmunoblastic T-cell lymphoma (AITL) is a very rare disease. Therefore, to identify the most prominent metabolic pathways on which AITL malignant Tfh cells depend, we utilized the pre-clinical AITL mouse model previously developed by us [9]. Closely resembling human AITL disease, murine CD4^+^ tumor cells expressed Tfh markers (PD-1, CXCR5) as their human counterparts, which are often defined as Tfh-like cells. The pool of CD4^+^ T cells in the plck-GAPDH tumors consisted of the PD-1 negative cells and the PD-1 high expressing cells (PD-1^high^) with the latter defined as the neoplastic cells in AITL malignancy. We therefore isolated PD-1^high^ CD4 T cells from mAITL tumors and WT CD4^+^ splenocytes and performed metabolomic analysis for these two populations. Firstly, metabolomics data principal component analysis showed a clear separation between the two cell populations (Fig. 1A). From the 70 metabolites detected demonstrating a significant change (Fig. 1B), 52 metabolites demonstrated a significant change > 1.5log^2^ between the PD-1^high^ CD4 mAITL cells and WT CD4 + splenocytes (Fig. 1C). We detected a significant downregulation or upregulation (> 1.5log^2^) in the mAITL cells versus WT CD4 + splenocytes for multiple lipids e.g. phosphatidylcholine (PC), sphingomyelin (SM) and lysophatidylcholine (LPC) (Fig. 1B-C). To confirm these changes in lipid metabolism in human AITL, we generated gene expression data for Tfh cells isolated from 8 different human AILT tumors and Tfh cells from healthy donors. C5/GOBP metabolic pathway analysis confirmed a significant upregulation in AITL and healthy donor Tfh subsets for lipid processes and lipid pathway activity (Fig. 1D). Lipid metabolism was also enriched for public data available for Tfh cells of 14 healthy donors. In contrast, this lipid pathways were not enriched in other CD4 T-cell subtypes such as central memory (Tcm), effector memory (Tem), naïve (Tn) and stem cell memory (Tscm) (Fig. 1D). Interestingly, regulatory T cells (Treg) also were marked by enrichment in lipid metabolic processes. Moreover, KEGG metabolic pathway analysis confirmed the high activity of lipid metabolism and biosynthesis in Tfh cells from AITL patient or healthy donors (Supplementary Fig. 1B).

Summarizing, in both mAITL and hAITL Tfh cells lipid metabolism was increased as compared to other T-cell populations.

### PD-1^high^ Tfh cells in the AITL mouse model rely on cytidine diphosphate (CDP)-choline metabolism

**Fig. 2:**
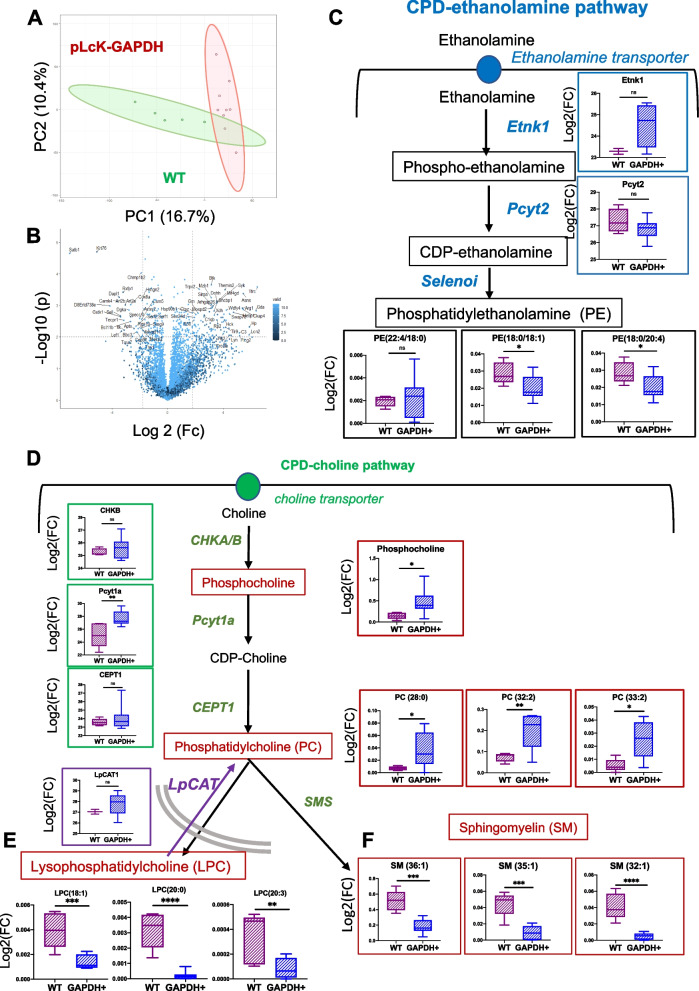
mAITL plck-GAPDH tumor cells rely on the CDP-choline pathway for increased phosphatidylcholine production. CD4 + PD-1^high^ cells were isolated from pLck-GAPDH mouse lymphoma and compared to WT CD4 + splenocytes for proteomic data analysis. Principle component analysis for the two groups in is shown in (**A**) and data are represented by a volcano plot in (**B**). **C** Expression data for the enzymes implicated in the CDP-ethanolamine pathway, Etnk1and Pcyt2 (blue quadrants) and the levels of phosphatidylethanolamine lipids (PE, black quadrants), the resulting end product of this pathway are shown for murine CD4 + PD-1^high^ cells tumor cells (GAPDH +) versus WT CD4 + splenocytes (**D**) Expression data for the three enzymes implicated in this CDP-choline pathway ChK, Pcyt1A, CEPTt1 (in green quadrant) and the levels of produced lipids in this pathway (phosphocholine and phosphatidylcholine (PC)) are shown in the red quadrants for murine CD4 + PD-1^high^ cells tumor cells (GAPDH +) versus WT CD4 + splenocytes. **E** Differences in expression levels of LPCAT the enzyme converting lysophosphatidylcholine (LPC) into PC is shown for both conditions in a blue quadrant. LPC levels are indicated in (**E**) Sphingomyelin levels (SM) are indicated for both conditions in (**F**). Data are represented as mean ± SD (Box and whisker plot representation, WT *n* = 5, GAPDH *n* = 8, *p < 0.05, **p < 0.01, ***p < 0.001, ****p < 0.0001, *ns* Not significant)

To reveal more specifically the lipid pathways involved in AITL, we performed proteomic analysis for the same T-cell populations, for which we generate metabolomics data: PD-1^high^ CD4 T cells from mAITL tumors and WT CD4 + splenocytes. Firstly, proteomics data principal component analysis showed a clear separation between the two cell populations (Fig. 2A) and multiple proteins showed significantly upregulated or downregulated expression levels (Fig. 2B). Interestingly, Fu et al. [15] showed quite recently that in healthy Tfh cells a specific lipid pathway, the CDP-ethanolamine pathway coordinated the expression and surface localization of CXCR5. This molecule is one of the hallmarks of Tfh cells and was shown to be stabilized by phosphatidylethanolamine (PE) at the membrane, the endproduct of the ethanolamine pathway. It has been stipulated by many studies that the malignant AITL CD4 + T cells strongly resembled healthy Tfh cells [16]. Therefore, we crossed metabolomics and proteomics data obtained for the mAITL malignant CD4 + T cells versus healthy murine CD4 + splenocytes to reveal the importance of the ethanolamine pathway in this malignancy. Ethanolamine kinase 1 (Etnk1) and phosphate cytidyl transferase 2 (Pcyt2) are the two main enzymes promoting de novo biosynthesis of PE (Supplementary Fig. 2 and Fig. 2C). We found that both Etnk1 and Pcyt2 were not significantly upregulated in mAITL CD4 + cells, while Selenoi providing the conversion into PE in the last reaction of the pathway, remained undetected. Interestingly, these data agreed with the significant reduction of several PE lipids (PE(22:4/18/0), PE(18:0/18:1), PE(18:0/20:4)) or no significant changes in PE lipids in mAITL CD4 + cells as compared to WT CD4 + splenocytes except for one PE lipid (Fig. 2C and Supplementary Fig. 3).

In parallel to this PE generating pathway, the related CDP-choline pathway is part of the Kennedy pathway (Supplementary Fig. 2 and Fig. 2D and Gibelini et al. [17]) that generates phosphatidylcholine (PC). PC is the main lipid structural element of cell membranes and is important for cell mitosis and signaling [18, 19]. Multiple cancers display an abnormal PC metabolism [20–22], which incited us to evaluate this lipid pathway in our mAITL CD4 + PD-1^high^ cells. The first step of this pathway is controlled by choline kinases (Chokα and Chokβ) which catalyze the phosphorylation of choline to generate phosphocholine (PCho). Chokα overexpression is associated with malignancy and recognized as a robust biomarker in cancer [23, 24]. We indeed detected a slight upregulation of choline kinase in the mAITL cells though not significant but the resulting metabolite phosphocholine was significantly increased (Fig. 2D). The enzyme Pcyt1a ensuring the next enzymatic step to obtain CDP-choline was also significantly augmented. Though Cept1 assuring the third step of the pathway namely converting CDP-choline into PC, was not upregulated a significant upregulation of PCs (PC(28:0), PC(32:2), PC(33:2); Fig. 2D) was detected and several PC lipids showed a tendency to increased levels though not significant (Supplementary Fig. 4; PC (36:5), PC (30:0), PC (34:2), PC (32:1), PC(40:9)). PC levels are also influenced by the Lands cycle, which is an alternative pathway of PC synthesis (Supplementary Fig. 2). Lpcat1, a key enzyme of this cycle, that converts lysophosphatidylcholine (LPC) into PC was found upregulated in mAITL cells (Fig. 2E), and was already reported to play a role in cancer pathogenesis and progression [25, 26]. This coincided with a very strong significant reduction in LPC lipids (LPC (18:1), LPC (20:0), LPC (20:3), LPC (18:0), LPC (16:0), LPC (20:4) in Fig. 2E and supplementary Fig. 5). Sphingomyelin (SM) is a ubiquitous structural component of mammalian cell membranes, and its cellular levels are regulated by both synthetic and catabolic pathways. In particular, the biochemical synthesis of SM occurs via the action of a sphingomyelin synthase (SMS), which transfers the phosphorylcholine moiety from PC onto the primary hydroxyl of ceramide, thus producing SM and diacylglycerol (DAG) (Jiang et al. [27], supplementary Fig. 2). Clearly SMS in mAITL CD4 + cells was not converting PC to SM since an up to threefold downregulation in these cells of several SM lipids was confirmed (Fig. 2F, SM(36:1), SM(35:1), SM(32:1)). Finally, PE can be converted into PC by the phosphatidylethanolamine N-methyltransferase (PEMT, Supplementary Fig. 2). However, no changes were found for this enzyme when comparing the mAITL CD4 and healthy CD4 + T cells.

Summarizing, the mAITL malignant Tfh cells are marked by an increase of CDP-choline pathway activity. Moreover, the lower SM levels and the fact that PCs were not used as a substrate to produce LPCs might be interpreted as the cause of PC accumulation in the mAITL Tfh-like cells possibly contributing to their malignancy.

### Patient AITL Tfh cell lipid metabolism relies on the CDP-choline pathway

**Fig. 3:**
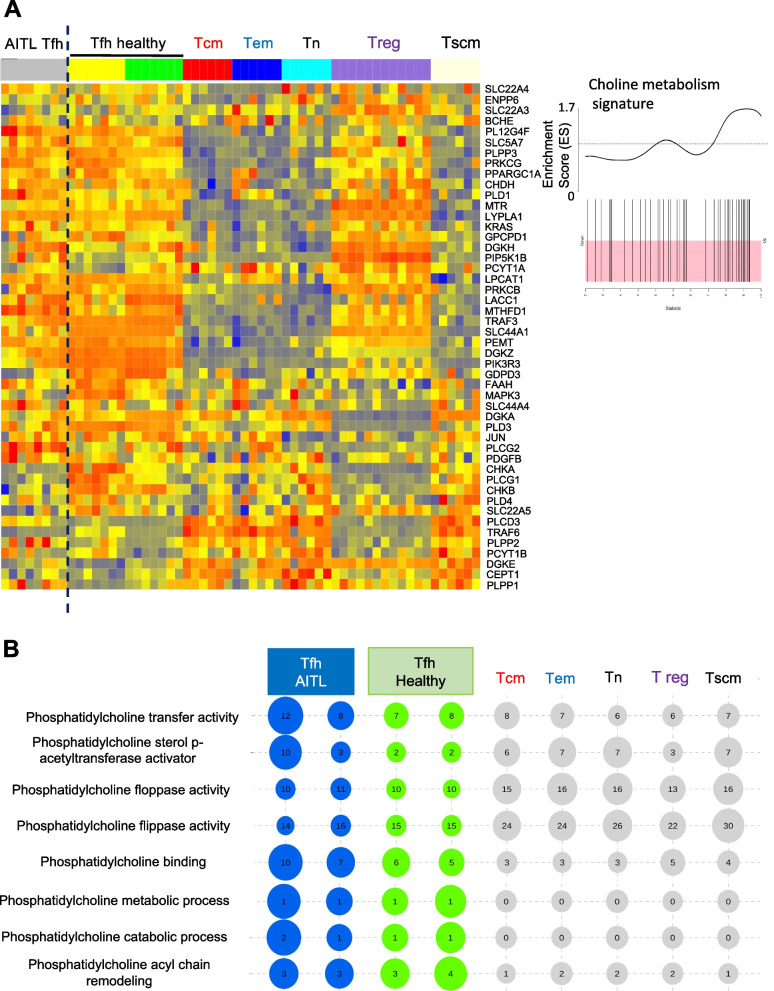
Human AITL malignant and healthy Tfh cells show increased choline pathway activity compared to other healthy CD4 T-cell subsets. **A** Heatmap for GSEA data of 48 genes implicated in the CDP-choline pathway for AITL patient Tfh cells (*n* = 8), healthy Tfh cells (*n* = 12), central memory T cells (Tcm) (*n* = 6), effector memory T cells (Tem) (*n* = 6), naive T cells (Tn) (*n* = 6), regulatory T cells (T reg) (*n* = 13) and stem memory T cell (Tscm) (*n* = 6). The corresponding GSEA for the choline pathway signature genes is indicated in (**A**, right panel). For all genes with enrichment score > 0 (black bars in the pink zone), expression is upregulated. Kolmogrov-Smimov (KS) test. **B** Reactome choline pathway analysis for GSEA data of the same T-cell populations as mentioned in (**A**) was performed. Bubble representation (Bubbles size and numbers represents the sample enrichment score (SES), *p*-values are indicated in Supplementary Table 1)

As mentioned above, in healthy human Tfh cells the CDP-ethanolamine pathway was revealed to be important for the Tfh differentiation and function. We indeed confirmed by analysis of GSEA data that Tfh cells from healthy donors showed an enrichment for the CDP-ethanolamine pathway gene signature as compared to the Tfh-like AITL cells. This was even more pronounced when comparing to central memory, effector memory, naïve and regulatory as well as stem cell memory T cells (Supplementary Fig. 6A). The key enzymes Etkn1 or Etkn2 and Pcyt2 were less expressed in Tfh AITL cells versus healthy donor Tfh (Supplementary Fig. 6A). Moreover, Reactome pathway analysis showed that four phosphatidylethanolomine related pathways were not highly enriched in AITL Tfh as compared to the healthy CD4 + Tfh and other T-cell subpopulations (Supplementary Fig. 6B). In agreement with the prominent use of the choline metabolic pathway by mAITL cells, heatmap GSE analysis revealed a statistically significant upregulation in AITL and healthy donor Tfh subsets for 48 genes coding for a choline metabolic signature (Fig. 3A). For this choline pathway signature GSEA was also enriched in public data available for Tfh cells of 14 healthy donors and regulatory T cells (Treg). This gene signature was not enriched though in other CD4 T-cell subtypes such as central memory (Tcm), effector memory (Tem), naïve (Tn) and stem cell memory (Tscm) T cells (Fig. 3A). Interestingly, GSEA analysis revealed a strong upregulation of choline transporters (SLC44A4, SLC44A1, SLC5A7) in AITL and healthy Tfh cells as compared to the other healthy CD4 + T-cell populations. Several other genes in this choline gene signature are highly expressed by AITL and healthy Tfh cells such as a phospholipase A2 (PLG2G4F) and choline dehydrogenase (CHDH). Although, CHKA or CHKB were not enriched in their expression in hAITL Tfh-like cells as compared to healthy Tfh or other CD4 + T-cell subtypes, the second step enzyme in choline metabolism CYT1A was strongly expressed in some AITL Tfh donors (Fig. 3A) equivalent to what we found for the mAITL Tfh cells (Fig. 2D). Additionally, LPCAT1, the enzyme converting LPC into PC was highly upregulated in its expression in as well AITL as healthy Tfh cells. Moreover, reactome pathway analysis confirmed in the hAITL Tfh subsets a strong enrichment of related phosphatidylcholine pathways (PC transfer, binding, floppase and flippase activity and PC metabolic process, Fig. 3B). This revealed the importance of an active PC lipid metabolism as well in AITL as in healthy Tfh cells.

**Fig. 4:**
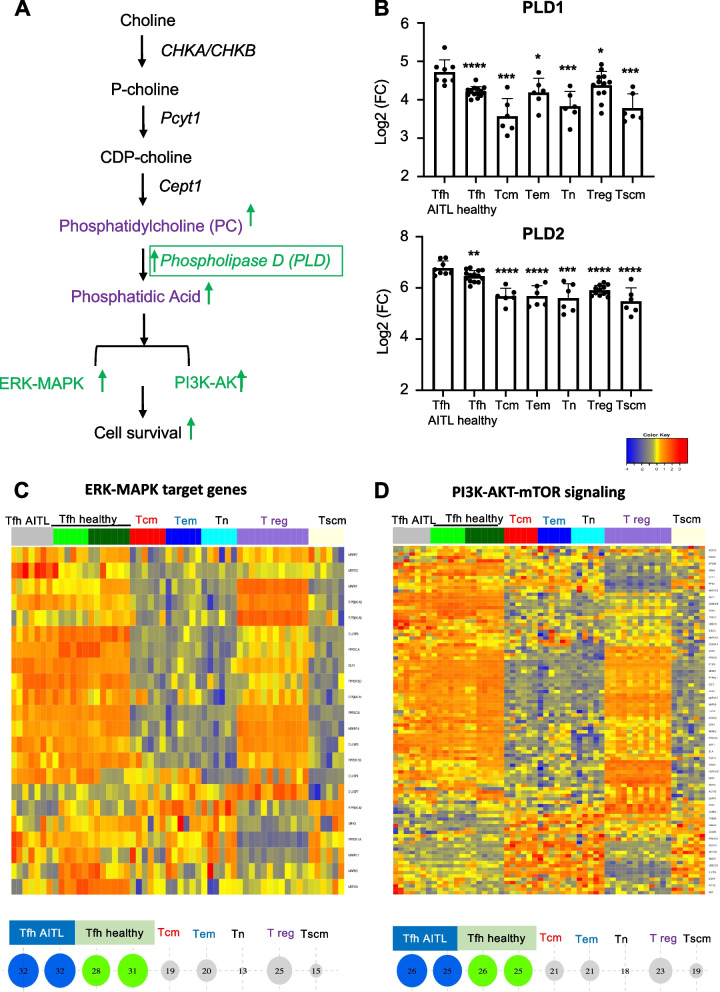
ERK-MAPK and the PI3K-AKT-mTOR pathways are induced in human AITL CD4 + PD1 + Tfh-like cells. **A** Schematic presentation of the pathway producing PC and its further conversion into PA (phosphatidic acid), which activates ERK-MAPK and PI3K-Akt pathways. **B** Expression levels of PDL1 and PDL2 for the different T-cell populations (AITL patient Tfh cells (*n* = 8), healthy Tfh cells (*n* = 12), central memory T cells (Tcm) (*n* = 6), effector memory T cells (Tem) (*n* = 6), naive T cells (Tn) (*n* = 6), regulatory T cells (T reg) (*n* = 13) and stem memory T cell (Tscm) (*n* = 6) (mean ± SD; *p < 0.05, **p < 0.01, ***p < 0.001, ****p < 0.0001). **C** Heatmap for GSEA data of 22 genes implicated in the ERK-MAPK target gene signature for AITL patient Tfh cells (*n* = 8), healthy Tfh cells (*n* = 12), central memory T cells (Tcm) (*n* = 6), effector memory T cells (Tem) (*n* = 6), naive T cells (Tn) (*n* = 6), regulatory T cells (T reg) (*n* = 13) and stem memory T cell (Tscm) (*n* = 6). Bubble representations for sample enrichment score (SES) of the ERK-MAPK target gene signature is shown (Bubbles size and numbers represents SES), *p*-value = 1.11e-024). **D** Heatmap for GSEA data of gene signature implicated in PI3K-Akt-mTOR signaling for the same T-cell subpopulations mentioned in (**C**). Bubble representations for sample enrichment score (SES) of the PI3K-Akt-mTOR signaling gene signature is shown (Bubbles size and numbers represents SES), *p*-value = 3.31e-017)

In the heatmap for GSEA analysis of a choline metabolism signature (Fig. 3A), the PC specific phospholipase, PLD1, was strongly expressed in AITL and healthy Tfh. Of note, choline kinase-activity is required for downstream production of phosphatidic acid (PA) by the PLD enzymes (Fig. 4A). It has been reported that PA, a mitotic second messenger derived from PC, is an important inducer of several Ras signaling pathways such as phosphoinositide 3-kinase (PI3K)/protein kinase B (AKT) signaling and extracellular signal regulated kinase (ERK)- MAP kinase (MAPK) signaling, confirmed to be implicated in (tumor) cell proliferation by Xiong et al. [28] (Fig. 4A). We confirmed that two phospholipases, PLD1 and PLD2, were significantly upregulated in human AITL Tfh cells compared to all the healthy CD4 + T-cell subsets including Tfh cells from healthy donors (Fig. 4B). Reactome GSEA analysis for the targets of both PI3K/AKT and ERK-MAPK signaling were highly enriched for both malignant and healthy Tfh cells compared to the other healthy CD4 + T-cell subsets (Fig. 4 C, D). This pointed to a connection between choline metabolism and PI3K/AKT and ERK-MAPK pathway induction as reported previously, which showed that inhibition of choline-kinase α suppressed simultaneously both pathways [29].

The aberrant expression of two proteins, Ras homolog family member A (RhoA) and Myc, have been reported to affect choline metabolism. Firstly, aberrant RhoA signaling was reported to activate choline kinase, which potentiated RhoA induced carcinogenesis [30]. This is of particular interest in the context of AITL since RhoA was found mutated in up to 70% of AITL patients [31] as well as in our mAITL mouse model (Supplementary Fig. 7A and B). As expected GSEA data analysis for Rho GPTase signaling and cycling showed a strong upregulation of RhoA activity (Supplementary Fig. 7C).

Secondly, Myc is a key oncogene that alters multiple tumor metabolic processes such as glycolysis, nucleotide and lipid synthesis [32, 33]. More precisely, Myc acts as a positive regulator of choline metabolism by inducing the transcriptional upregulation of Pcyt1a, the rate limiting enzyme of PC biosynthesis [34]. GSEA analysis revealed indeed a strong enrichment of a Myc target gene signature in human AITL and healthy Tfh cells, as compared to effector memory, central memory, naïve and stem cell memory T cells, indicating its possible interference with choline metabolism (Supplementary Fig. 8).

Summarizing, the hAITL malignant Tfh cells were marked by an increase of CDP-choline pathway activity similar to their healthy counterparts, coinciding with increase in RhoA and Myc activity, known inducers of choline metabolism. Moreover, PI3K/AKT and ERK-MAPK signaling pathways, shown to be induced by the end product of the PC generating pathway were increased in hAITL neoplastic cells.

### Inhibition of lipid metabolism by etomoxir increased survival of mAITL preclinical model

Lipid metabolism is strongly dependent on fatty acid oxidation (FAO) and FAO fuels the production of metabolites needed to synthesize lipids such as PCs mentioned above [35].

FAO is dependent on carnitine palmitoyltransferase 1 (CPT1a), a key enzyme in the transport of fatty acids across the mitochondrial inner membrane (Supplementary Fig. 9A) [36].

**Fig. 5:**
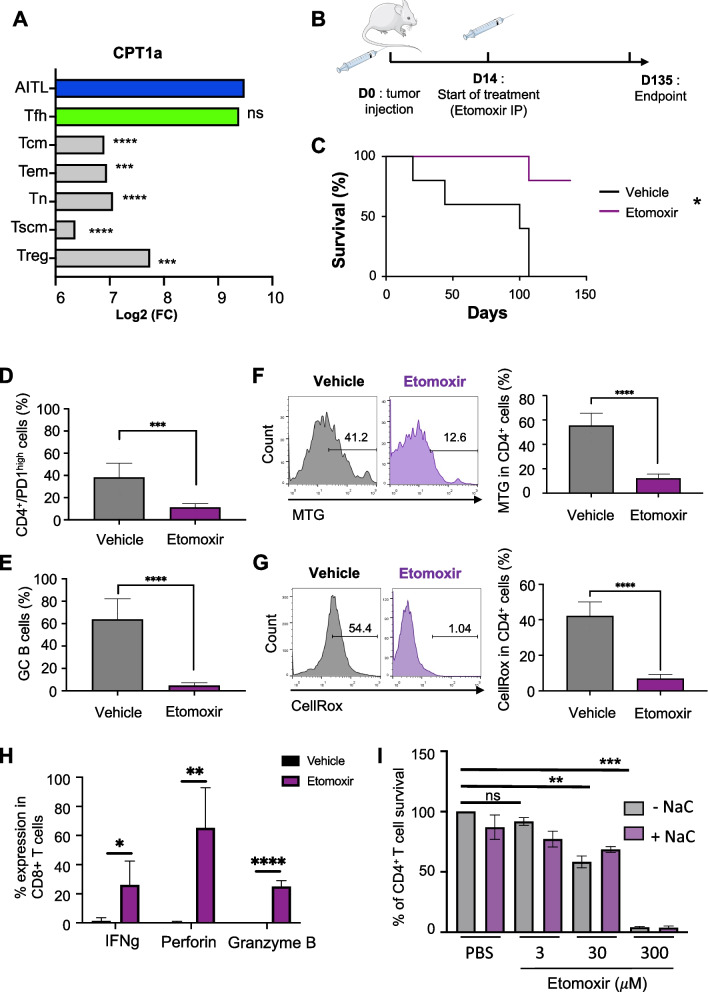
Inhibition of CDP-choline pathway by etomoxir prolonged the survival of the mAITL preclinical model. **A** Expression levels of CPT1a (inhibited by etomoxir) for the different T-cell populations (AITL patient Tfh cells (*n* = 8), healthy Tfh cells (*n* = 12), central memory T cells (Tcm) (*n* = 6), effector memory T cells (Tem) (*n* = 6), naive T cells (Tn) (*n* = 6), regulatory T cells (T reg) (*n* = 13) and stem memory T cell (Tscm) (*n* = 6) (mean ± SD, ***p < 0.001, ****p < 0.0001, ns: non-significant). **B** Splenic lymphoma cell from plck-GAPDH mice were injected intravenously into recipient NSG mice (*n* = 11), which were treated with vehicle (*n* = 5) of with the Cept1 inhibitor etomoxir (*n* = 6). Survival curves for both mice groups are shown in (**C**). Mice were sacrificed at humane endpoint or 140 days post transplant (*p < 0.05, Mantel-Cox test). **D** FACS analysis of percentage of CD4 + PD1.^high^ cells per total CD4 + T cells in the spleen of the indicated treatment groups at sacrifice; data are summarized in the histogram (mean ± SD, Vehicle. *n* = 5, Etomoxir *n* = 6), ***p < 0.001). **E** FACS analysis of percentage of GC B cells (GL-7 + CD95 +) on total B cells in the spleen of the indicated treatment groups at sacrifice; data are summarized in the histogram (mean ± SD, Vehicle *n* = 5, Etomoxir *n* = 6, ****p < 0.0001). **F** FACS analysis of CD4 + T cells stained for mitochondrial content by Mitotracker green (MTG) in the spleen of the indicated treatment groups at sacrifice; data are summarized in the histogram (mean ± SD, vehicle *n* = 5, Etomoxir *n* = 6, ****p < 0.0001). **G** FACS analysis of CD4 + T cells stained for ROS content by CellROX probe in the spleen of the indicated treatment groups at sacrifice; data are summarized in the histogram (mean ± SD, vehicle *n* = 5, etomoxir *n* = 6, ****p < 0.0001). **H** Splenocytes isolated from etomoxir or control-treated mAITL engrafted NSG mice were activated for 6 h with PMA/ionomycin in presence of golgi-stop, then surface stained for CD8 followed by intracellular staining for INFγ, perforin and granzyme B and analysed by FACS (mean ± SD, *n* = 4, *p < 0.05,**p < 0.01, ****p < 0.0001). (I) mAITL CD4 + T cells were treated with etomoxir at the indicated doses in the absence (-NAC) or presence of N-Acetyl-L-cysteine (+ NAC; 2 mM) for 96 h followed by FACS analysis for the CD4 + T-cell survival (mean ± SD, *n *= 3, ***p < 0.001, ****p < 0.0001, *ns* non significant)

CPT1a expression was found indeed increased significantly in hAITL Tfh cells and healthy Tfh cells as compared to all other of T cell subsets (Fig. 5A). In addition, the downstream enzyme CTP2 was found to be upregulated, pointing towards increased FAO activity in hAITL cells [37] (Supplementary Fig. 9B). It has been reported that impairing FAO by etomoxir can halt tumor progression in both solid cancers and hematological malignancies [35, 38–40]. Of note, more in particular, etomoxir was shown to induce apoptosis in in vitro models of acute myeloid leukemia (AML), accompanied by a significant reduction in phosphatidylcholine (PC) and its metabolism [41]. Therefore, we chose to interfere with total lipid metabolism in the mAITL preclinical model by blocking FAO. CPT1a is the rate-limiting enzyme in long chain FAO. We therefore choose etomoxir, which inhibits at low doses CPT1a, an enzyme that ensures the carnitine-dependent FA transport across the inner mitochondrial membrane [41]. Etomoxir blocks in this way the entry of FA into mitochondria and avoids their β-oxidation, forcing the cells to use glucose for their energy requirements [42].

We assessed the effects of inhibiting FAO by etomoxir on tumor growth in vivo in our mAITL transplant model (Supplementary Fig. 9A and Fig. 5B). Etomoxir treatment resulted in significantly increased survival of mAITL tumor-engrafted mice (Fig. 5C). Additionally, a significant decrease in CD4 + PD-1^high^ and associated GC B cells in the etomoxir treated group was detected (Fig. 5D-E). Previously, we have shown that the CD4 + PD-1^high^ cells have a high quantity of mitochondria as also a high ROS content (unpublished data). Here, we demonstrated that the residual CD4 + T cells in the etomoxir group were low in ROS content coinciding with a decreased mitochondrial content as compared to vehicle treated mice, resembling healthy CD4 T cells (Fig. 5F-G). Additionally, this intervention with FAO metabolism also induced a strong immune response confirmed by INFγ, granzyme B and perforin production by cytotoxic CD8 + T cells in the mAITL preclinical model (Fig. 5H). Importantly, etomoxir was shown not only to inhibit CPT1 at low concentrations (3 μM etomoxir) but also the complex I of the mitochondrial electron transport chain at higher concentration (> 100 μM etomoxir, Supplementary Fig. 9A and Raud et al. [43]). To distinguish between these two activities of the inhibitor, we treated murine lymphoma cells with increasing doses of etomoxir and determined the CD4 + PD1^high^ cell death 3 days after treatment (Fig. 5I). Although at the lowest etomoxir concentration (3 μM) mAITL malignant cells were not significantly affected, a tenfold higher concentration (30 μM), which still not majorly inhibits the mitochondrial complex I, induced about 40% of cell death (Fig. 5I). The highest concentration (300 μM), which inhibits the electron transport chain [43], wiped out the CD4 + PD1^high^ cells completely. Another off-target effect of etomoxir is the promotion of ROS production [44]. As long as cells can resolve excessive ROS by intrinsic pathways this will not effect cell fitness or survival. Our results showed that addition of N-Acetyl-L-cysteine (NAC), a ROS scavenger, did not revert the effect of etomoxir on CD4 + PD1^high^ cell survival (Fig. 5I). Indeed, ROS neutralization by NAC in the presence of etomoxir did not increase malignant T cell survival (Fig. 5I) indicating that ROS-mediated toxicity was not a major effect induced by etomoxir.

Finally, we evaluated additionally another more specific FAO inhibitor, ranolazine [45], which blocks acetyl Co-A production from fatty acids in the mitochondria [46]. We choose ranolazine as an alternative to etomoxir because this FAO inhibitor most probably shows less off-effects. A second advantage is that ranolazine has been approved in the United States and Europe as a treatment for angina [47], in contrast to etomoxir, which cannot be used in the clinic since it induced high liver toxicity in patients [48]. Treatment with escalating ranolazine doses induced significant death of malignant CD4 + T cell in mAITL biopsies (Supplementary Fig. 10). Ranolazine already exerts at low doses (10 μM) malignant T cell killing, indicating its potency as an FAO inhibitor (Supplementary Fig. 10). Etomoxir, in contrast, shows no significant malignant T cell killing at a low doses reported to inhibition FAO (3 μM). Only a superior dose (30 μM) is sufficient to induce AITL CD4 + T cell death (Fig. 5I).

In summary, these data put forward that interfering with global lipid metabolism by inhibiting FAO permitted to increase survival of the AITL mouse model.

### mAITL lymphoma recipient mice respond to Chok inhibition by reduction in CD4 + PD-1 ^high^ T cells, which was confirmed in AITL biopsies

**Fig. 6:**
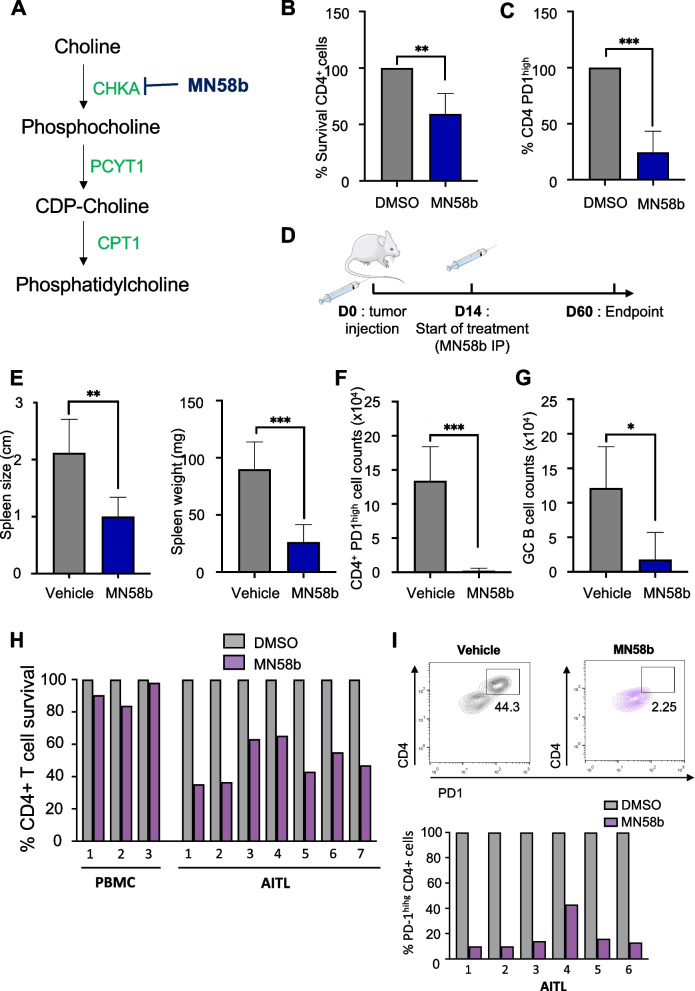
Inhibition of Chokα preferentially eliminated CD4 + PD1^high^ cells from the tumors in mAITL preclinical model and from AITL patient biopsies in vitro. **A** Schematic representation of the CDP-choline pathway with the step inhibited by MN58b indicated. (**C**) Effect of Chokα inhibitor MN58b treatment on mAITL lymphoma cells in vitro. The survival of the CD4 + T cells (**B**) and the % of residual CD4 + PD1^high^ tumoral cells (**C**) are indicated. Percentages are normalized to corresponding vehicle-treated cells set at 100%. **D** Splenic lymphoma cells from plck-GAPDH mice were injected intravenously into recipient NSG mice (*n* = 10), which were treated with vehicle (*n* = 5) or MN58b (*n* = 5) by IP injection. Mice were sacrificed at 60 days post-transplant. **E** Spleen size and weight are shown for the indicated treatment groups at sacrifice. CD4 + PD1^high^ (**F**) and GC B (**G**) cell counts in the spleen for the indicated treatment groups at sacrifice. Data are summarized in the histograms (mean ± SD, Vehicle: *n* = 5, MN58b: *n* = 5; *p < 0.05, **p < 0.01, ***p < 0.001). **H** Effect of Chokα inhibitor (MN58b) treatment (72 h) on the CD4 + T cell survival of LN biopsies of 7 different AITL patients compared to CD4 + T cells in PBMCs from healthy donors as control. Percentages are normalized to corresponding vehicle-treated (DMSO) control cells set at 100%. **I** FACS analysis of percentage of CD4 + PD1^high^ cells on total CD4 + T cells of LN biopsies of 6 different AITL patient biopsies after 72 h of MN58b or Vehicle (DMSO) treatment. A representative FACS plot is shown for *n* = 6 and individual AITL donors are represented in a histogram

Next, we wanted to evaluate the effect of blocking the CDP-choline pathway using a more specific inhibitor of choline metabolism. We decided to inhibit choline kinase alpha (Chokα), the enzyme responsible for the first step in this pathway, by a specific inhibitor MN58b, which is a choline mimetic drug (Fig. 6A and references [24, 49]). Firstly, we evaluated MN58b mediated inhibition of Chokα on mAITL biopsies in vitro. A strong reduction in number of surviving CD4 + T cells was induced and additionally the neoplastic CD4 + PD-1^high^ cells were primarily affected (Fig. 6B-C). This result incited us to treat the preclinical mAITL mice with MN58b (Fig. 6D). Due to the toxic side-effects of MN58b on the digestive tracts of the mice, which prevented the establishment of a survival curve, the mice had to be sacrificed earlier at a humane endpoint (data not shown). Inhibition of Chokα led to significant reduced spleen size and weight. Importantly, in the spleens of MN58b treated mAITL mice the malignant CD4 + PD-1^high^ cells were almost completely wiped out (Fig. 6F) accompanied by a reduction in GC B cells (Fig. 6G).

Healthy PBMCs and cells isolated from AITL patient biopsies were treated with MN58b, a choline competitor for Chokα. Upon treatment healthy peripheral blood CD4 + T cells were not affected by inhibition of the first step in the choline metabolic pathway, while a strong reduction in the survival of human CD4 T AITL T cells was detected (Fig. 6H). Importantly, in particular the CD4 + T cells expressing the highest levels of PD-1 in the AITL samples were almost completely wiped out (Fig. 6I).

Concluding, inhibition of the CDP-choline lipid pathway by the choline mimetic drug MN58b resulted in a strong reduction of the mAITL Tfh cells in the spleen and halted disease in the preclinical mAITL mouse model. Moreover, hAITL biopsies treated with the same drug significantly reduced the CD4 + PD-1^high^ lymphoma cells.

## Discussion

Thanks to our previously generated AITL mouse model [9], which mimics human AITL closely in its pathological features, immune and genetic phenotypes and signaling pathway activation, we revealed by integrating metabolomics and proteomics data that the neoplastic Tfh lymphoma cells were strongly dependent on the CDP-choline pathway, one of the branches of the Kennedy pathway. We confirmed that neoplastic AITL Tfh cells from patients also sustained active choline metabolism. Interfering with general lipid metabolism by treatment with the FAO inhibitors, etomoxir, even increased survival of the mAITL preclinical mouse model. Another FAO inhibitor, ranolazine, a clinically accepted drug, showed equivalent effects in vitro on mAITL biopsies. Further specific inhibition of the CDP-choline pathway in the mAITL preclinical mice model at its first enzymatic step (Chokα/Chokβ) resulted in selective elimination of the neoplastic CD4 + PD-1^high^ T cells in vivo. Moreover, the neoplastic CD4 + PD-1^high^ T cells in human biopsies were highly sensitive to specific inhibition of the choline metabolic pathway and were promptly induced into cell death.

### Importance of lipid metabolism in cancer

Changes in lipid metabolism are of major importance during tumor development [50, 51]. Lipids provide a storage of energy for the cell and serves as a major component of the cellular membranes but also as a source of signaling molecules. Obviously, the high demand for lipid synthesis of cancer cells for both growth and survival is not surprising. Neither that many mutated oncogenes play an important role in lipid synthesis regulation by changing for example the activity of lipid enzymes [52]. One of those enzymes, Chokα, when overexpressed was shown sufficient to establish malignant transformation suggesting that choline is of utmost importance for lipid-mediated proliferation of cancer cells and since long considered a therapeutic target [24, 52–55]. CHKA encodes for two splice variants of Chokα. CHKB encodes for Chokβ, which has 60% similarity to CHKA in sequence. While Chokα protein is more selective for the choline substrate, Chokβ has a higher affinity for ethanolamine. Chokα alterations were frequently detected in breast cancer, colon cancer, lung cancer, prostate cancer, ovary, pancreatic, skin and brain cancers [21, 24, 49, 52, 53]. Importantly, Chokα levels are not only increased in solid tumors but also significantly increased in human diffuse large B cell lymphoma (DLBCL), multiple myoloma, and Burkit’s lymphoma [23, 56]. In agreement, increased levels of choline transporters were detected in DLBCL and HL [57, 58]. For these B cell malignancies, Chokα inhibition revealed to be a good treatment option in preclinical models and provided a rational basis to bring these inhibitors further to the clinic.

### Upregulation of choline metabolism in B and T cell malignancies

Moreover, the expression of the oncogene MYC is often augmented or activated in B cell malignancies and acts for example in DLBCL as a positive regulator of choline metabolism through augmented expression of Pcyt1a, a rate limiting enzyme in this pathway [34]. Similarly, we found in that the hAITL Tfh-like cells showed strong MYC activity, possibly modulating the PC biosynthesis. Indeed, inhibition of Pcyt1a exhibited anti-lymphoma activity on glioblastoma and DLBCL in vitro and in vivo [34]. As in DLBCL lymphomas, we found in our mAITL mouse model that the neoplastic lymphoma T cells showed a significant upregulation of Pcyt1a, which might represent in addition to Chokα, another target in choline metabolism for anti-cancer treatment. As in B cell malignancies, Xiong et al. [28] showed that choline levels in T-cell lymphoma cells were systematically augmented and caused by Chokα up-regulation. Additionally, Mariotto et al. [59] reported that Chokα protein levels were also elevated in acute lymphoblastic leukemia (T-ALL). Inhibition or genetic silencing of Chokα in these T-cell malignancies strongly affected their proliferation and survival, which we confirmed here for AITL lymphoma T cells. This confirmed, as we have shown for mAITL cells, that other malignant T cells rely on choline metabolism for their tumorigenicity.

### Choline metabolism and AKT/ERK activity in T cell lymphoma

Xiong et al. [28] also showed increased Chokα-induced AKT/ERK activity in T-cell lymphoma cells which was correlated with Myc oncoprotein expression. Moreover, Chokα and Chokβ were found to regulate Akt, a critical mediator in metabolic pathways and cell survival through phosphorylation of Akt at Ser473, activating Akt in a PI3K-dependent way [29]. Here we indeed showed that the PI3K-AKT-mTOR axis is active in hAITL neoplastic cells (Fig. 4D). This might indeed be a consequence of Chokα action directly but also due to further metabolic conversion of the end product of the choline pathway, PC, as shown in glioma development [60], into phosphatidic acid (PA), known as an inducer of several Ras signaling pathways such as PI3K/AKT and ERK-MAPK signaling [21, 54]. Indeed, PLD1 and PLD2 enzymes responsible for PC conversion into PA were highly upregulated in hAITL Tfh cells compared to other healthy CD4 T-cell subsets and even to a significantly higher extent as compared to healthy Tfh cells. In accordance, we also found the ERK-MAPK target genes upregulated in hAITL neoplastic cells (Fig. 4C).

### Chokα inhibition as an anti-cancer therapy

For long now, Chokα has been recognized as a potential therapeutic target for human cancers, which has led to the development of a variety of Chokα inhibitors of which the first prototype was hemicholinium-3 (HC-3), a choline mimetic drug [24, 53]. The compound used in our study MN58b is derived from HC-3. While both drugs inhibited cancer cell proliferation through apoptosis [24, 53], MN58b was more specific in its Chokα inhibition and was in contrast to first generation drugs, proved not toxic for healthy tissues. In our study we clearly showed a reduction in lymphoma development upon MN58b administration in the mAITL mouse model. However, long-term 2-weekly administration of MN58b in vivo, was nevertheless toxic in our mAITL developing mice. One Chokα inhibitor called TCD-717 was evaluated in a phase I clinical trial in patients (NCT01215864) with advanced solid tumors but results are up to date are not available, which unfortunately impedes us to gain insight in efficacy versus safety of this treatment. More effective and well-tolerated Chokα inhibitors are a required and a focus of current interest.

### Therapeutic interference with FAO in mAITL cells

Etomoxir is the best-known inhibitor of CPT1, a rate limiting enzyme in the FAO cycle. It was already used in different cancers in order to block FAO metabolism [61]. Presti et al. [41] showed that AML cells reduced their levels of PCs significantly upon etomoxir treatment. We demonstrated that etomoxir administration exerted an anticancer activity on mAITL cells in vivo and in vitro. While at high doses etomoxir as a side-effect also inhibit complex I of the mitochondrial electron transport chain (ETC) [43], we confirmed that even at etomoxir levels lower than the one required for ETC inhibition, mAITL cells were sensitive to this drug and still induced cell death. Moreover, recent studies have shown that etomoxir in addition to inhibiting CPT1, an essential enzyme in FAO metabolism, can also increase ROS production to toxic levels in cancer cells [44]. Neutralizing ROS with the ROS scavenger NAC was not able to revert the effect of etomoxir on mAILT tumor cells suggesting this was not the major way of action exerted by etomoxir. The tumor microenvironment though might also be affected by changes in FAO activity or ROS levels. Interestingly, though FAO was believed to be important for regulatory (Treg) and memory T cells (Tmem) differentiation [62, 63]. Raud et al. (2018) demonstrated through genetic invalidation of CPT1 that long chain FAO is largely dispensable for as well Treg and Tmem cell activation, differentiation and function. Therefore, Treg and Tmem differentiation and function in the tumor microenvironment should not be affected at low etomoxir concentration (3 μM). However, at higher doses, excessive ROS levels induced by etomoxir [43], that cannot be resolved by these T cells might lead to ROS mediated toxicity affecting Treg and Tmem cell survival and functions [64, 65]. Importantly, we have shown that etomoxir treatment in vivo of the mAITL lymphoma did not inhibit CD8 effector function since they exerted high cytotoxicity.

Another drawback is that etomoxir is not approved for therapeutic intervention in patients because it was associated with liver toxicity in a clinical trial [48]. Therefore, other drugs that inhibit FAO metabolism such as ranolazine, which is approved by the FDA and the European Medicines Agency for treatment of angina might be a better option [47]. Importantly, ranolazine induced cell death of malignant CD4 T cells in biopsies from our mAITL preclinical mouse model by blockage of acetyl Co-A production from fatty acids in the mitochondria [46]. Since ranolazine is effective in killing AITL malignant cells it suggests that possibly this drug could be repositioned for treatment of AITL patients, which lack effective treatment. This will need further preclinical testing.

### Ethanolamine versus choline lipid metabolism

The Kennedy pathway for lipid generation was recently reported to be involved in Tfh cells immunity. More specifically, Fu et al. [15] identified the CDP-ethanolamine pathway for de novo synthesis of PE, which functioned as a selective post-transcriptional regulator of Tfh cell differentiation by promoting and stabilizing CXCR5 expression at the T-cell surface. AITL neoplastic T cells have numerous features similar to Tfh cells such as high expression of PD-1, CXCR5, Bcl-6 and CXCL-13 [66]. We confirmed for healthy donor Tfh cells an enrichment of the CDP-ethanolamine pathway signature (supplementary Fig. 6), which was not as pronounced though in hAITL Tfh-like cells coinciding with a lower CXCR5 expression. Additionally, the mAITL Tfh-like cells were not mainly relying on the CDP-ethanolamine pathway but in contrast we found upregulation of enzymes and metabolites in the parallel choline pathway, confirmed by an increase in several PC lipids (Fig. 2D). This also holds for patient AITL Tfh-like cells. However, the choline metabolic signature was also highly enriched in healthy Tfh cells. The Fu et al. [15] study though demonstrated by genetic invalidation of the major players in this pathway (CHKA, CHKB, PCYT1A/B or CEPT1) that choline metabolism was not required for healthy Tfh cell generation. In AITL malignancy, this is clearly not the case since inhibiting this pathway had a major effect on lymphoma T-cell survival in vitro and in vivo in the mAITL preclinical mouse model and human AITL in vitro.

PC lipid accumulation in AITL, a phenomenon detected in multiple cancers [21, 22], partially might be attributed to an alternative lipid generating pathway, the Lands cycle, which results in Lpcat1 mediated conversion of LPC into PC lipids. Lpcat1 modulation can play an important role in cancer pathogenesis and progression [25, 26] and we found this enzyme also significantly upregulated in the mAITL and hAITL neoplastic Tfh cells which suggest that Lpcat1 inhibition might also be a possible novel treatment option.

## Conclusion

In conclusion, we have demonstrated that neoplastic AITL cells are highly dependent on lipid metabolism. In particular the lipid pathway producing phosphatidylcholine, a major cell membrane component, was found to be essential for their survival and proliferation. Targeting lipid metabolism through etomoxir-mediated FAO inhibition or by inhibition of the choline metabolic pathway using a Chokα inhibitor nearly eradicated all AITL Tfh in our mAITL model and from AITL patient biopsies. This emphasized the therapeutic value of interfering with these lipid metabolic pathways for the treatment of AITL patients, which have no access to efficient anti-cancer drugs up to now. Etomoxir though lacks specificity and is not approved for clinical use due to major liver toxicity. Ranolazine, another more specific FAO inhibitor, already approved for treatment of angina might offer a better alternative. Unfortunately, available Chokα inhibitors also have toxic side-effect and are not approved for use in patients. Hopefully, this research might encourage screening for safe drugs interfering with these pathways and contribute the development of new treatments for AITL.

### Supplementary Information

**Additional file 1. **

## Data Availability

The data and material generated in this study are available upon requestion from the corresponding author (els.verhoeyen@unice.fr). Affymetrix data generated by us is available from Gene Expression Omnibus (GEO) (GSE232609, confidential Token for access: gnsjgkyozjulnmn). Other data for comparison were obtained from GEO (GSE61697, GSE65010, GSE66384 and GSE71566). GSE19069, GSE58445) and E-TABM-783 (https://www.ebi.ac.uk/biostudies/arrayexpress /studies/E-TABM-783). Proteomics and metabolomics are available within supplementary data files.
