# Supplementary material for: Inhibition of choline metabolism in an angioimmunoblastic T-cell lymphoma preclinical model reveals a new metabolic vulnerability as possible target for treatment
Source: J Exp Clin Cancer Res. 2024 Feb 6;43:43. doi: 10.1186/s13046-024-02952-w (PMC10845598; doi:10.1186/s13046-024-02952-w)
Supplement: Supplementary file 1 — Additional file 1. [file 13046_2024_2952_MOESM1_ESM.pdf]

A.

Pathway	p-value
Membrane lipid metabolic process	7.41873265284243e-35
Membrane lipid catabolic process	3.15712659065963e-31
Membrane lipid biosynthetic process	5.88470625336672e-37
Lipid storage	2.23063958421603e-25
Lipid phosphorylation	1.39426601452112e-09
Lipid oxydation	7.74901370007308e-11
Lipid metabolic process	1.4812415751695e-34
Cellular lipid metabolic process	1.50930748123647e-31

B.

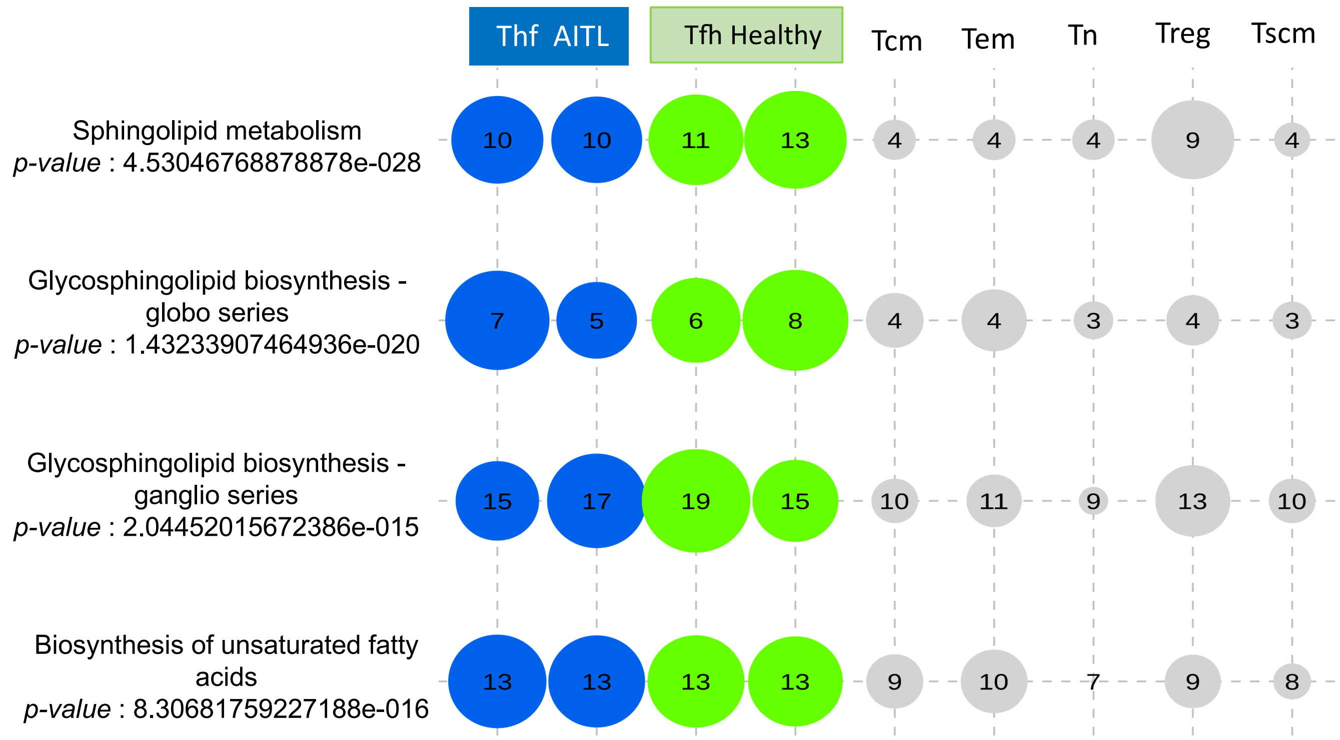

Supplementary Figure 1. Upregulation of lipid metabolism in hAITL Tfh cells versus healthy CD4+ T cell subsets.
(A) p-values for the Reactome pathway analysis in Figure 1D.
(B) Specific lipid pathway analysis from GSEA data of isolated Tfh cells from AITL lymphoma versus healthy Tfh cells and versus public RNA-seq data from healthy Tfh cells (Tfh_public), central memory (Tcm), effector memory (Tem), naïve (Tn), regulatory (Treg) and stem cell memory (Tscm) was performed using the KEGG database. p-values are indicated.

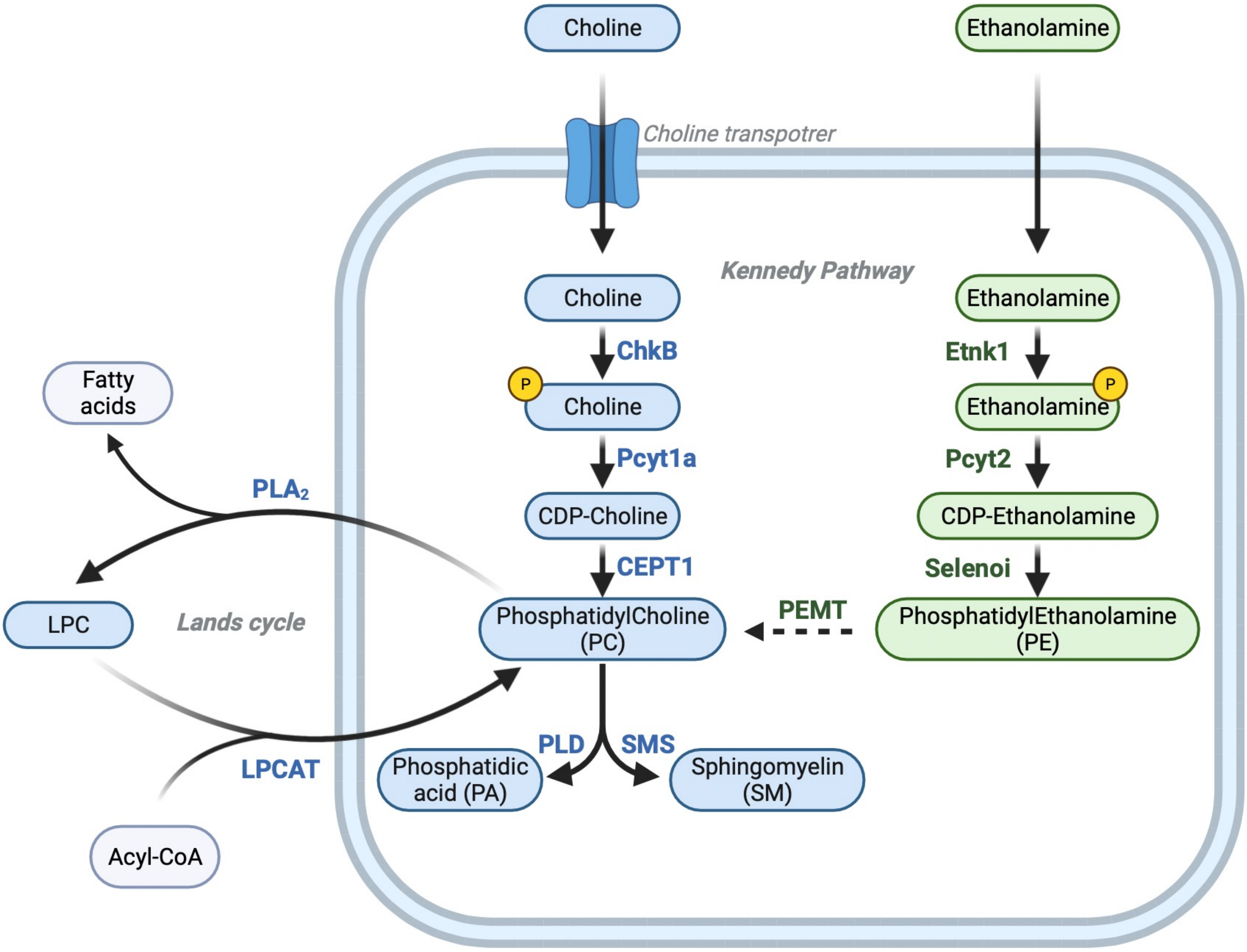

Supplementary Figure 2. Schematic representation of the Kennedy pathway comprising the CPD-ethanolamine pathway and the parallel CPD-choline pathway and also the different pathways influencing the PC levels.
Chk: choline kinase; pcyt1a: phosphate cytidyl transferase 1; CEPT1: Choline/Ethanolamine Phosphotransferase 1; PLD; PC specific phospholipase D; SMS: sphingomyelin synthase; LPCAT: lysophosphatidylcholine acyltransferase 1 PLA2 LPC: lyosphosphatidylcholine; Etnk1: ethanolamine kinase; Pcyt2 phosphate cytidyl transferase 2;. PEMT: phosphatidylethanolamine N-methyltransferase.

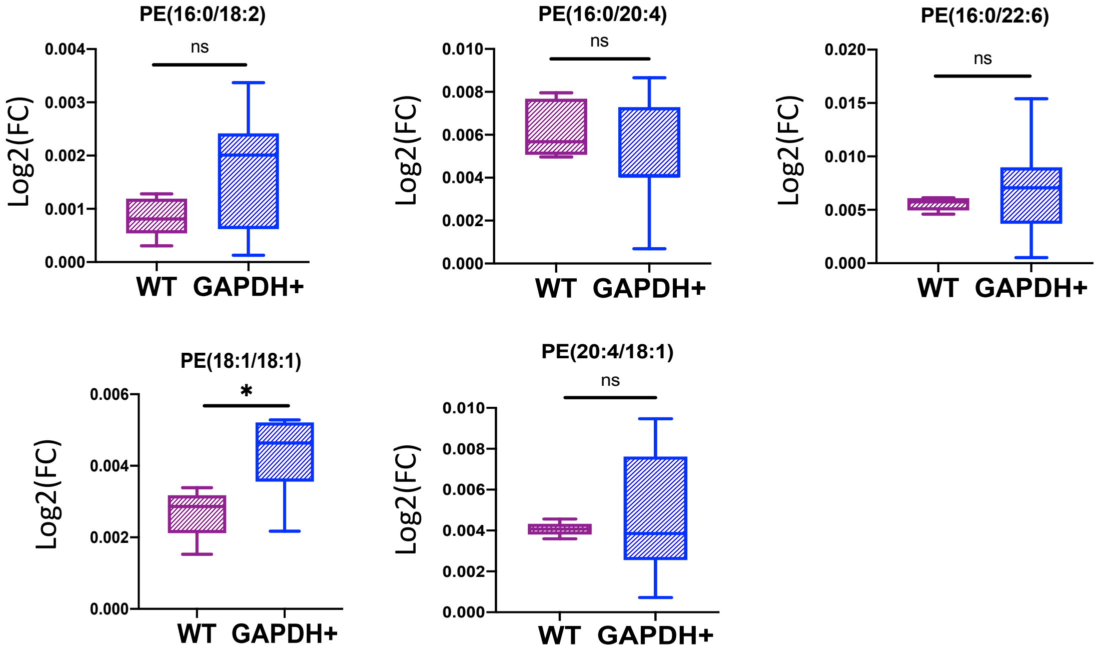

Supplementary Figure 3: In CD4+ PD-1^{high} mAITL cells, CPD-ethanolomine pathway activity does not results in higher levels of phosphatidyl-ethanolamine (PE) lipids

CD4+ PD-1^{high} cells were isolated from pLck-GAPDH mouse lymphoma and compared to WT CD4+ splenocytes for metabolite analysis. The levels of phosphatidylethanolamine lipids (PE), the product of this pathway are shown for murine CD4+ PD-1^{high} tumor cells (GAPDH+) versus WT CD4+ splenocytes (mean±SD, WT n=5; GAPDH n=8; *p<0.05).

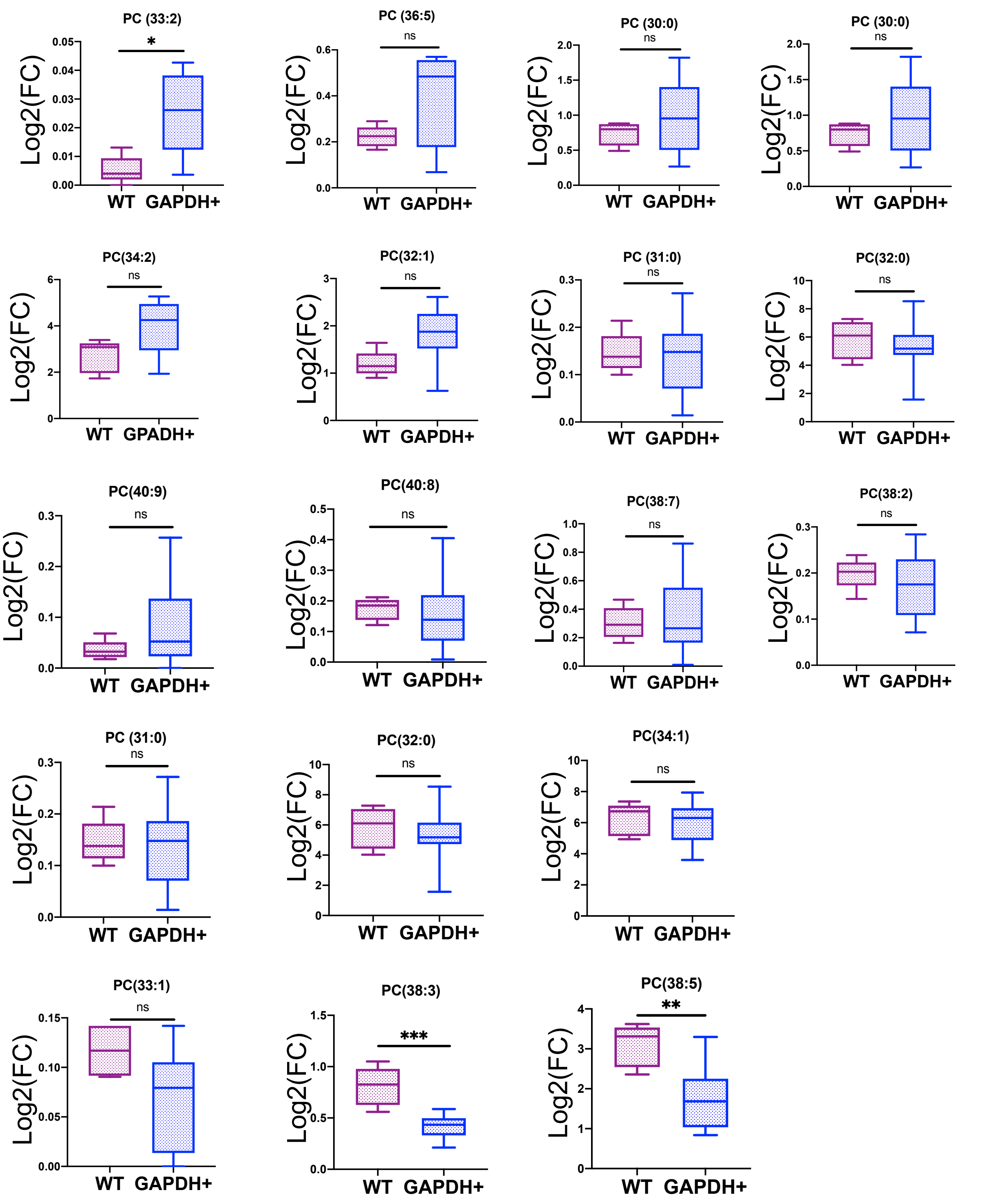

Supplementary Figure 4: In CD4+ PD-1^{high} mAITL cells, CPD-choline pathway activity results in higher levels of several phosphadidyl choline (PC) lipids
(A) CD4+ PD-1^{high} cells were isolated from pLck-GAPDH mouse lymphoma and compared to WT CD4+ splenocytes for metabolite analysis. The levels of phosphatidylcholine lipids (PC), the product of this pathway are shown for murine CD4+ PD-1^{high} cells tumor cells (GAPDH+) versus WT CD4+ splenocytes (mean±SD, WT n=5; GAPDH n=8; *p<0.05, **p<0.01, ***p<0.001).

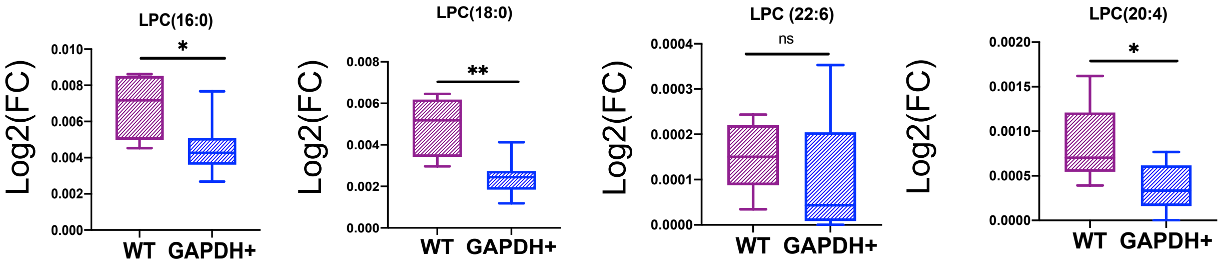

Supplementary Figure 5: CD4+ PD-1^{high} mAITL cells do not convert PC into LPCs through the Lands cycle

CD4+ PD-1^{high} cells were isolated from pLck-GAPDH mouse lymphoma and compared to WT CD4+ splenocytes for metabolite analysis. The levels of detected lysophosphatidylcholine lipids (LPC), the product of the Lands cycle pathway are shown for murine CD4+ PD-1^{high} cells tumor cells versus WT CD4+ splenocytes (mean±SD, WT n=5; GAPDH n=8; *p<0.05, **p<0.01).

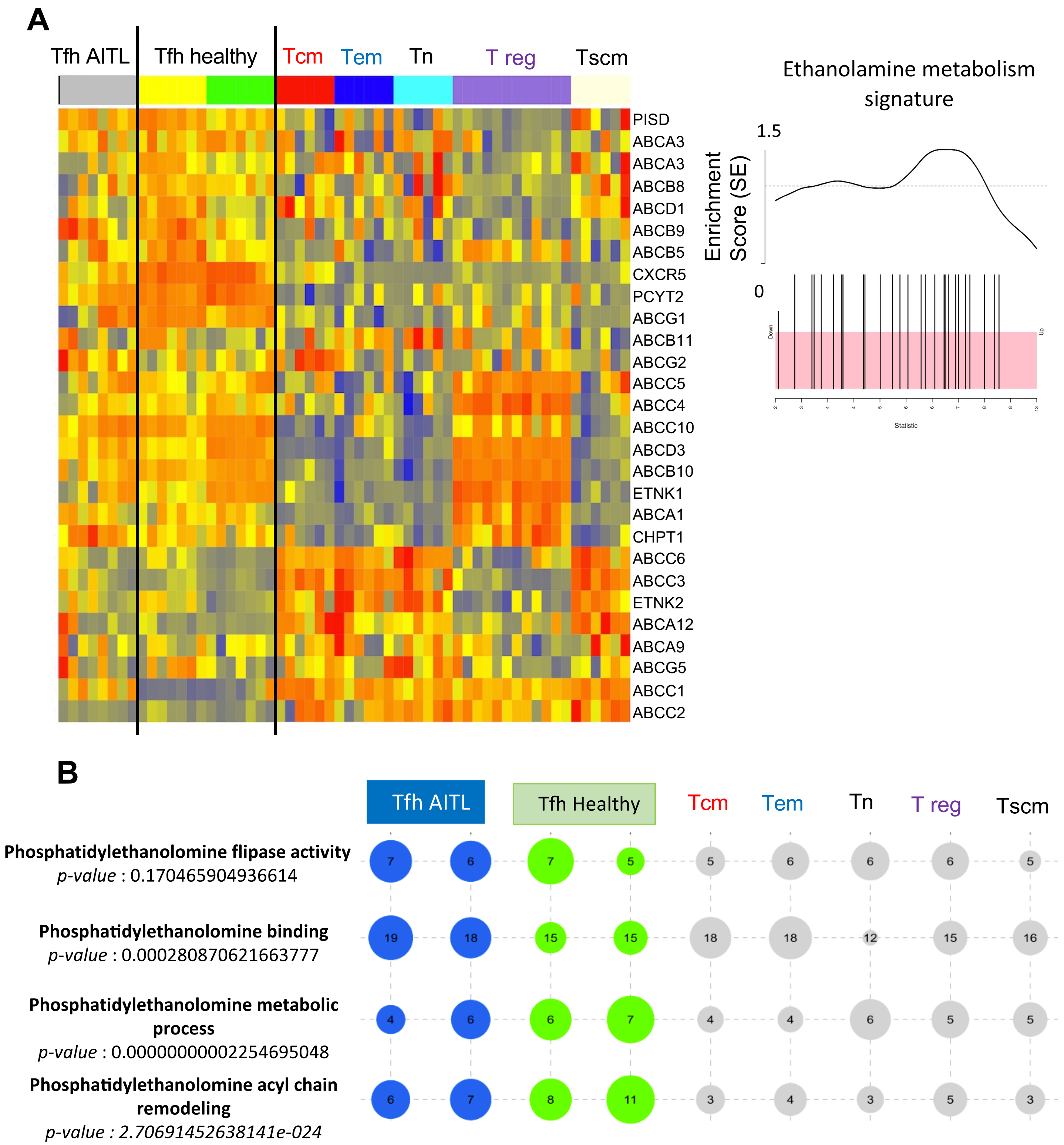

Supplementary figure 6: The CDP-ethanolamine pathway signature is less enriched in AITL patient Tfh cells compared to healthy Tfh cells

(A) Heatmap for GSEA data of 28 genes implicated in the ethanolamine pathway for AITL patient (n=8), healthy Tfh cells (n=12), central memory T cells (Tcm) (n=6), effector memory T cells (Tem) (n=6), naive T cells (Tn) (n=6), regulatory T cells (T reg) (n=13) and stem memory T cell (Tscm) (n=6). The corresponding GSEA for the ethanolamine pathway signature genes indicated in (A) is shown at the right. For all genes with enrichment score > 0 (black bars in the pink zone), expression is upregulated. Kolmogrov-Smimov (KS) test.

(B) Ethanolamine pathway analysis for GSEA data of the same T cell populations as mentioned in (A) was performed. Bubble representation (Bubbles size and numbers represents the sample enrichment score (SES); p-values are indicated).

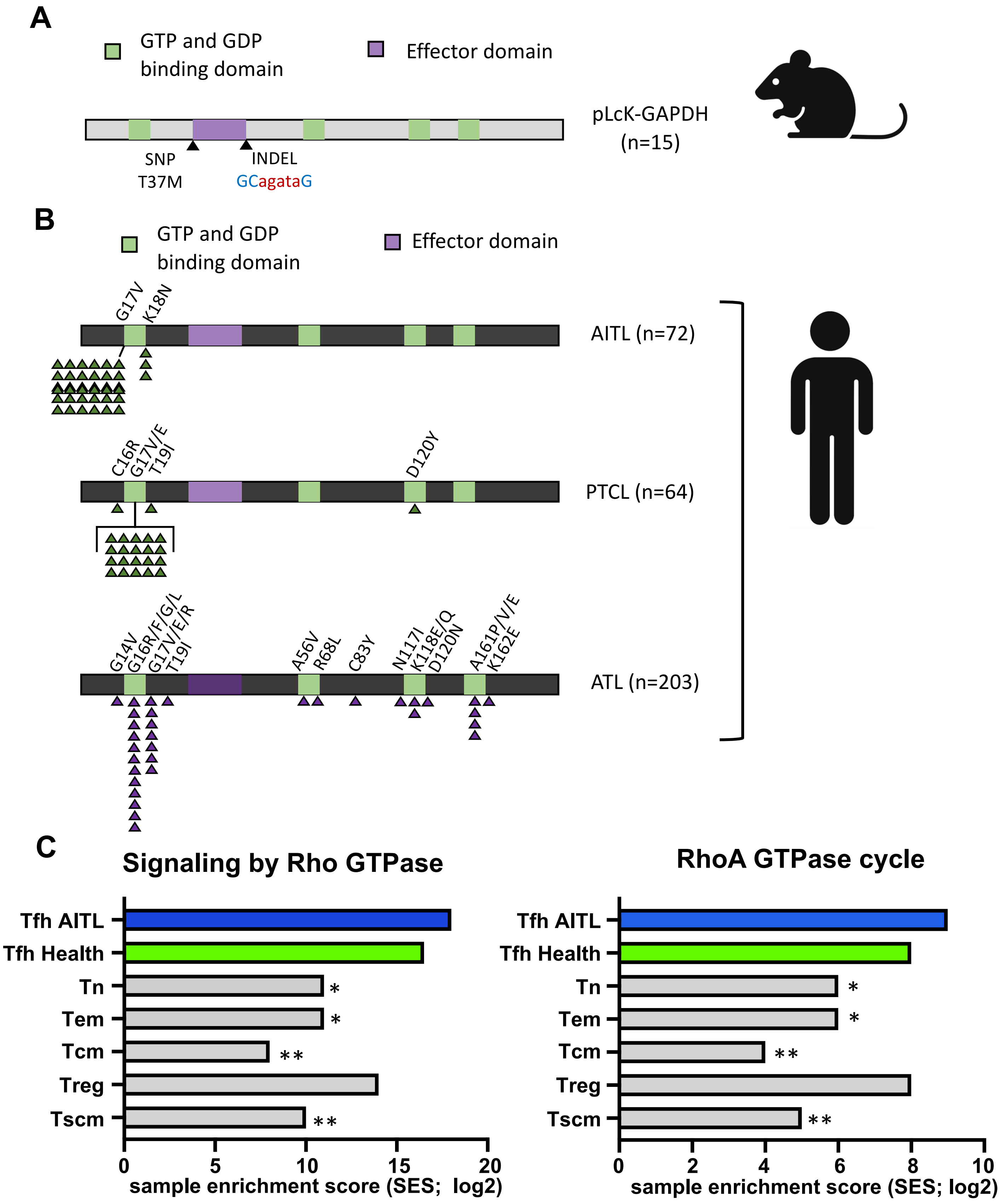

Supplementary figure 7: RhoA mutations in mAITL and human AITL Influence RhoA activity

(A) Mutations in RhoA detected for the Plck-GAPDH lymphoma cells. (B) Mutations in RhoA detected in malignant cells for AITL patients, peripheral T cell lymphoma (PTCL) and acute T cell lymphoma (ATL). (C) GSEA data for AITL patient (n=8), healthy Tfh cells (n=14), central memory T cells (Tcm) (n=6), effector memory T cells (Tem) (n=6), naive T cells (Tn) (n=6), regulatory T cells (T reg) (n=13) and stem memory T cell (Tscm) (n=6). Bars represents the sample enrichment score (SES).

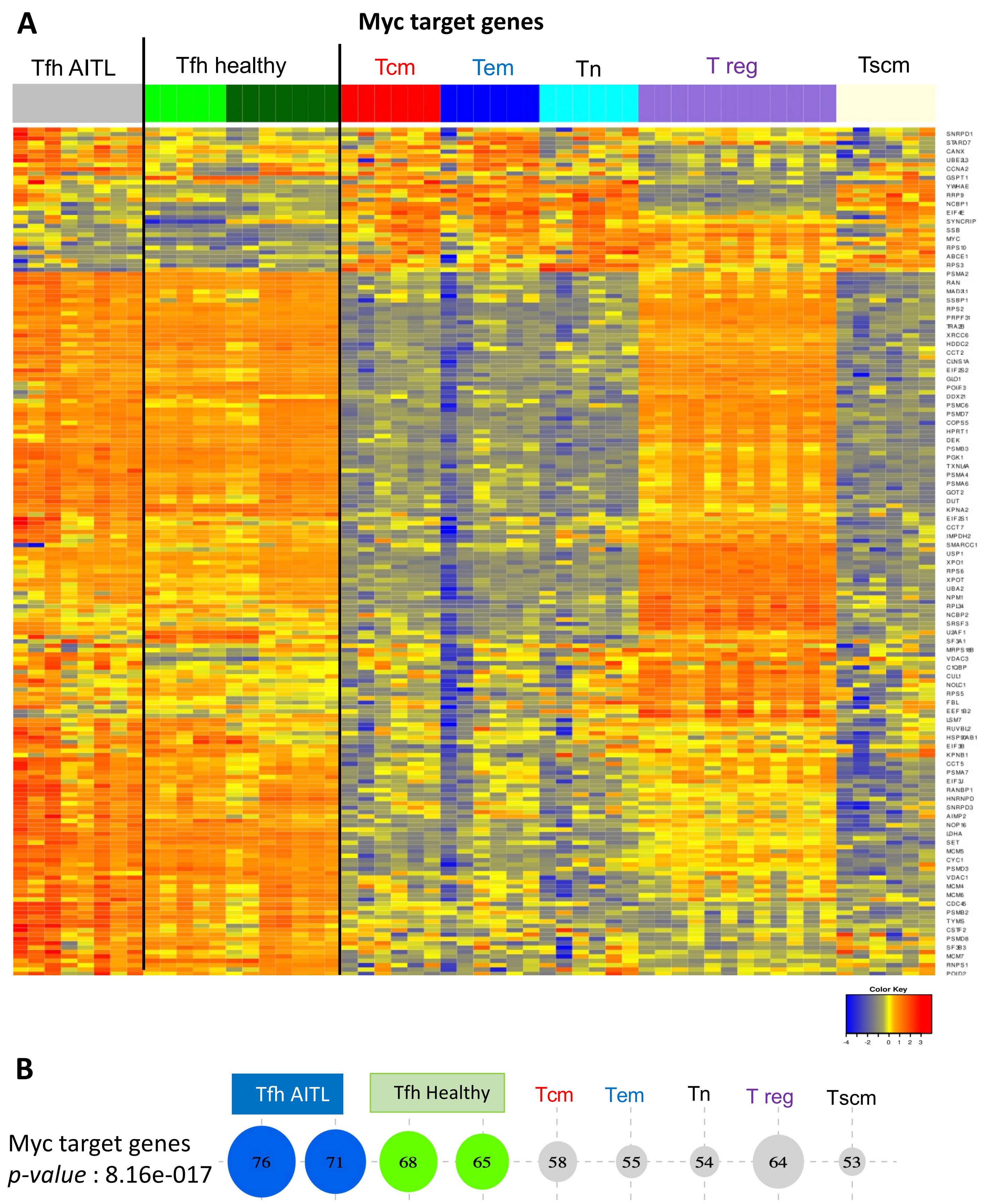

Supplementary Figure 8: Myc is highly active in hAITL and healthy Tfh cells compared to other CD4 T cell subsets

(A) Heatmap for GSEA data of a Myc target gene signature for AITL patient (n=8), healthy Tfh cells (n=12), central memory T cells (Tcm) (n=6), effector memory T cells (Tem) (n=6), naive T cells (Tn) (n=6), regulatory T cells (T reg) (n=13) and stem memory T cell (Tscm) (n=6). (B) Bubble representations for sample enrichment score (SES) of the myc target gene signature is shown (Bubbles size and numbers represents SES); p-value is indicated).

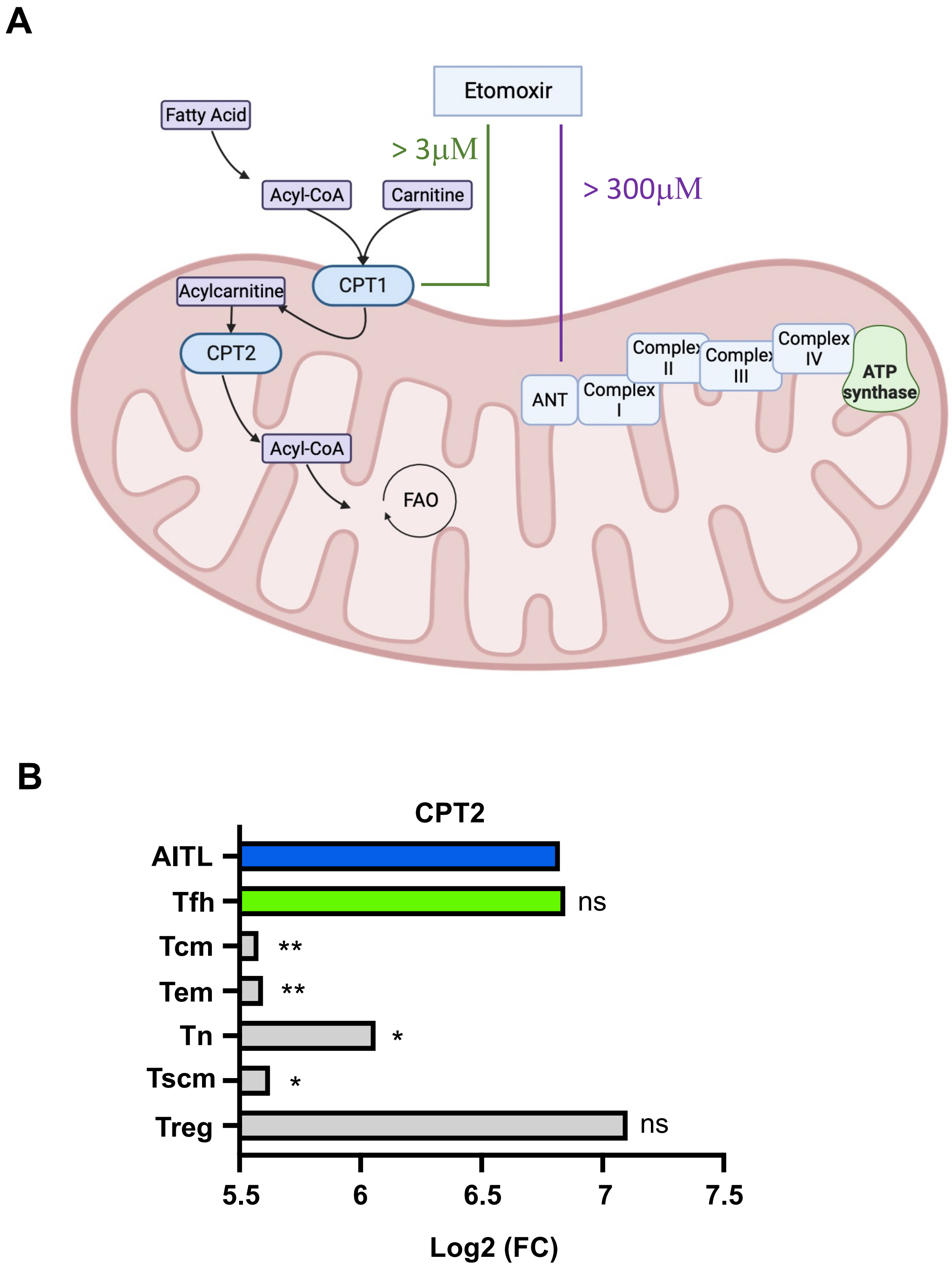

Supplementary figure 9 : Enzymes implicated in FAO are highly upregulated in AITL Tfh as compared to other heatly T cell subsets

(A) Schematic representation of the inhibition of CPT1a by Etomoxir at low doses, the rate limiting step in fatty acid oxydation or at high doses of the mitochondrial respiration by inhibition of complex I of the electron transport chain. (B) Expression levels of CPT2 for the different T cell populations (AITL patient Tfh cells (n=8), healthy Tfh cells (n=12), central memory T cells (Tcm) (n=6), effector memory T cells (Tem) (n=6), naive T cells (Tn) (n=6), regulatory T cells (T reg) (n=13) and stem memory T cell (Tscm) (n=6) (mean±SD; $^{**}p<0.01$, $^{*}p<0.05$, ns: non-significant).

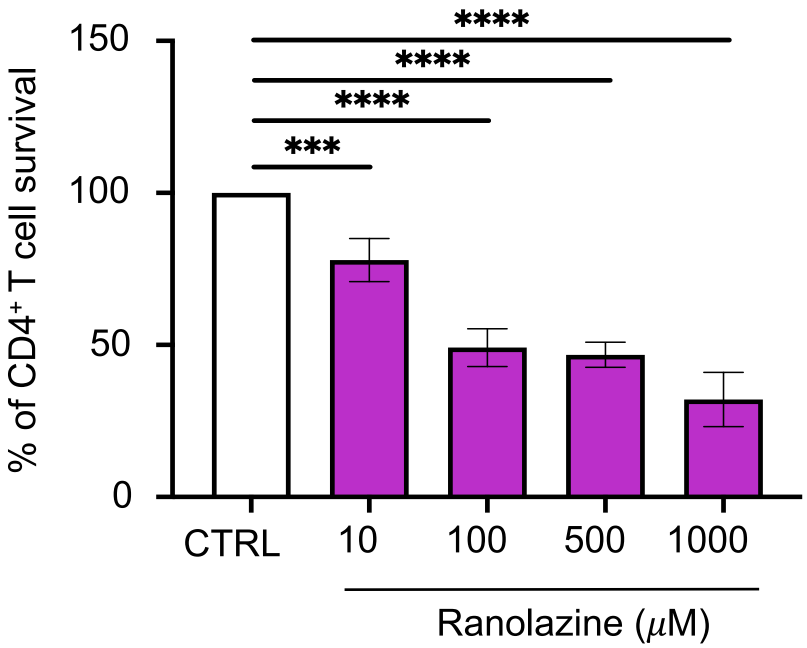

Supplementary Figure 10: The FAO inhibition by ranolazine induced cell death of the malignant CD4+ T cells in mAITL biopies.

Murine AITL bioposies were treated with ranolazine at the indicated doses for 96h followed by DAPI staining and FACS analysis for the CD4+ T-cell survival (mean±SD, n=4; ***p<0.001****p<0.0001).

Supplementary Table 1 Choline pathway analysis for GSEA data for the different T cell populations in Figure 3A. The p-values for the Enrichment scores represented by bubble graphs in Figure 3B.

Pathway	p-value
Phosphatidylcholine transfer activity	0.000522658061519948
Phosphatidylcholine sterol o-acyltrasferase activator activity	0.00000000000293529364
Phosphatidylcholine floppase activity	0.00000086577041837332
Phosphatidylcholine flippase activity	0.00000000000088979565
Phosphatidylcholine binding	1.9438240568018e-16
Phosphatidylcholine metabolic process	1.90434576144961e-21
Phosphatidylcholine catabolic process	5.9069345976615e-20
Phosphatidylcholine acyl chain remodeling	4.00395763704897e-23

Supplementary material **and methods**

Tumor transplantation into NOD/SCIDgc-/- (NSG) mice

NOD/SCIDgc-/- mice (NSG) were obtained from the Jackson Laboratory (#005557) and bred and maintained under pathogen-free conditions at the local animal facility (C3M, INSERM U1065, Nice, France). Single cell suspensions from the spleen of aged plck-GAPDH mice (>18 months) with splenomegaly were intravenously injected (i.v.) into 6- to 8-week old NSG mice (1-2x10^7 splenocytes per mouse). Recipient mice were sacrificed at endpoint (> 10% weight loss or palpable splenomegaly). The different hematopoietic organs (spleen, liver and lymph nodes) were isolated and single cell suspension were prepared for FACS analysis (see below).

Isolation of primary mouse T cells

$CD4^+$ T cells from the spleen of WT or plck GAPDH mice were isolated by negative selection using FITC coupled antibodies: anti-CD19 (Miltenyi, #130-102-494) anti-B220 (Miltenyi, #130-110-845), anti-CD8b (Miltenyi, #130-111-710), anti-Ter119 (Miltenyi, #130-102-257), anti-NK1.1 (BD Pharmingen, #553164), anti-CD49b (BD Pharmingen, #553857), anti-Ly 6C (BD Pharmingen, #553127), anti-CD122 (BD Pharmingen,# 554452) and anti-CD11c (BD Pharmingen, #553801), followed by anti-FITC microbead (Miltenyi, #130-048-701) incubation and AutoMACS isolation according to manufacturer's instructions (Miltenyi). The CD4+ negative T cells fraction was then incubated with anti-PD1-PE (Miltenyi; #130-111-800) followed by anti-PE microbead (Miltenyi, #130-105-639) incubation and AutoMACS isolation according to manufacturer's instructions (Miltenyi) to obtain the AITL CD4+ $PD1^{high}$ and CD4+ PD- cells for further proteomic and metabolite analysis.

Proteomics analysis

Protein extraction and enzymatic digestion

For each biological replicate, approximately 2E6 sorted cells were pelleted in PBS, flash frozen and stored at -80°C. Protein extraction was performed in 8M urea in 50 mM ammonium bicarbonate (ABC) followed by sonication in a Bioruptor (Diagenode, 15 cycles, 30s on, 30s off, high mode). Proteins were reduced in 10 mM dithiothreitol for 20 min at room temperature

(RT) and alkylated with 50 mM iodoacetamide for 30 min at RT. Digestion was carried out in 8M urea in 50 mM ABC for 2 h at RT with LysC (Wako Fujifilm, 1:100 w/w), after which the digestion buffer was diluted to final 2M urea with 50 mM ABC and trypsin (Promega, 1:100 w/w) was added for overnight digestion at RT. Digestion was stopped by adding acetonitrile (ACN) to 2% and trifluoroacetic acid (TFA) to 0.3% and the samples were cleared by centrifugation for 5 min at 10 000g. Peptides were purified by loading the cleared supernatant into C18 StageTips (1), and eluted with 80% ACN, 0.5% acetic acid. Finally, the elution buffer was eliminated by vacuum centrifugation and the purified peptides were dissolved in 2% ACN, 0.5% acetic acid, 0.1% TFA for single-shot LC-MS/MS measurements.

LC-MS/MS analysis

Peptides were separated on an EASY-nLC 1200 HPLC system (Thermo Fisher Scientific) coupled online via a nanoelectrospray source (Thermo Fisher Scientific) to a Q Exactive HF mass spectrometer (Thermo Fisher Scientific).
Peptides were loaded in buffer A (0.1% formic acid) into a 75 µm inner diameter, 50 cm long column packed in-house with ReproSil-Pur C18-AQ 1.9 µm resin (Dr. Maisch HPLC GmbH), and eluted over a 150 min linear gradient of 5-30% buffer B (80% ACN, 0.1% formic acid) at a 250 nl/min flow rate. The Q Exactive HF was operated in a data-dependent mode with the Xcalibur software (Thermo Scientific), with a survey scan range of 300-1,650 m/z, resolution of 60,000 at 200 m/z, maximum injection time of 20 ms and AGC target of 3e6.
The ten most abundant ions with charge 2 to 5 were isolated with a 1.8 m/z isolation window and fragmented by higher-energy collisional dissociation (HCD) at a normalized collision energy of 27. MS/MS spectra were acquired with a resolution of 15,000 at 200 m/z, maximum injection time of 55 ms and AGC target of 1e5. Dynamic exclusion was set to 30 s to reduce repeated sequencing.

LC-MS/MS data analysis

MS raw files were processed with the MaxQuant software v.1.6.7.0.The integrated Andromeda search engine (2) was employed to search spectra against the Mouse UniProt database) and a database of common contaminants (247 entries) to identify peptides and proteins with a false discovery rate of < 0.01. Enzyme specificity was set as “Trypsin/P” with a maximum of 2 missed cleavages and 7 amino acids minimum length. N-terminal protein acetylation and methionine oxidation were set as variable modifications, while cysteine carbamidomethylation was set as a fixed modification. Match between runs was used to transfer identifications across samples based on mass and normalized retention times, with a matching time window of 0.7 min and an alignment time window of 20 min. Label-free protein quantification (LFQ) was performed with the MaxLFQ algorithm (3) with a minimum required peptide ratio count of 1. Data analysis was performed using the R statistical computing environment. Data were filtered by removing proteins only identified by site, reverse hits, and potential contaminants. After log2 transformation of LFQ intensities, biological replicates were grouped. For statistical analysis, missing data points were replaced by imputation from a normal distribution with 0.3 width and 1.8 downshift, and a two-sided two-samples t-test was used to evaluate significant protein intensity changes. The mass spectrometry proteomics data have been deposited to the ProteomeXchange repository with the dataset identifier PXD046786 (Reviewer’s access: Username = reviewer_pxd046786@ebi.ac.uk and Password: YfCVbSXr)

Metabolite analysis

Sample preparation was performed as described previously (4,5) and is detailed in the supplementary material. Briefly, 1 mL of methanol at − 40 °C and 1 mL of cold water were added to the cell suspension for metabolite extraction. After centrifugation, the supernatant was dried and dissolved in 50 µL of methanol/water (50:50 v/v), and 10 µL was injected into the LC-HRMS system.

LC-HRMS analysis was performed using an UPLC Ultimate WPS-3000 system (Dionex, Germany) coupled to a Q-Exactive mass spectrometer (Thermo Fisher Scientific, Bremen,

Germany) and operated in positive (ESI+) and negative (ESI−) electrospray ionization modes (analysis for each ionization mode) was used for this analysis. Liquid chromatography was performed using a Phenomenex Kinetex 1.7 µm XB – C18 column (150 mm x 2.10 mm) maintained at 55°C. Two mobile phase gradients were used. The gradient was maintained at a flow rate of 0.4 mL/min over a runtime of 20 min. Two different columns were used to increase the metabolic coverage. Accordingly, a hydrophilic interaction liquid chromatography (HILIC) column (150 mm x 2.10 mm, 100 Å) was also used. During the full-scan acquisition, which ranged from 58 to 870 m/z, the instrument operated at 70,000 resolution (m/z = 200). As required for all biological analyses, the pre-analytical and analytical steps of the experiment were validated by findings of Quality Control (QC) samples (mix of all the samples analyzed). Coefficients of variation [CV% = (the standard deviation/ mean) x 100], were calculated from

all metabolites data and metabolites having a CV in QCs >30% were excluded from the final dataset.

A targeted analysis was applied on the samples, based on a library of standard compounds (Mass Spectroscopy Metabolite Library (MSML®) of standards, IROA TechnologiesTM). The following criteria were followed to identify the metabolites: (1) retention time of the detected metabolite within ± 20 s of the standard reference, (2) exact measured of molecular mass of the metabolite within a range of 10 ppm around the known molecular mass of the reference compound, and (3) correspondence between isotopic ratios of the metabolite and the standard reference. The signal value was calculated using Xcalibur® software (Thermo Fisher Scientific, San Jose, CA) by integrating the chromatographic peak area corresponding to the selected metabolite.

Statistical analysis were performed using MetaboAnalyst computational platform. The data analysis was first conducted using a multivariate approach, principal component analysis (PCA) to discriminate groups. For univariate analysis, statistical analysis was performed using student's T-test).

Metabolomics data have been deposited to the EMBL-EBI MetaboLights database with the identifier MTBLS9070. The complete dataset is available at https://www.ebi.ac.uk/metabolights/MTBLS9070.

References

1. Rappsilber J, Mann M, Ishihama Y. Protocol for micro-purification, enrichment, pre-fractionation and storage of peptides for proteomics using StageTips. Nat Protoc. 2007;2:1896–906.
2. Cox J, Neuhauser N, Michalski A, Scheltema RA, Olsen JV, Mann M. Andromeda: a peptide search engine integrated into the MaxQuant environment. J Proteome Res. 2011;10:1794–805.
3. Cox J, Hein MY, Luber CA, Paron I, Nagaraj N, Mann M. Accurate proteome-wide label- free quantification by delayed normalization and maximal peptide ratio extraction, termed MaxLFQ. Mol Cell Proteomics. 2014;13:2513–26.

4. Madji Hounoum B, Blasco H, Nadal-Desbarats L, Diémé B, Montigny F, Andres CR, et al. Analytical methodology for metabolomics study of adherent mammalian cells using NMR, GC-MS and LC-HRMS. Anal Bioanal Chem. 2015;407:8861–72.
5. Madji Hounoum B, Mavel S, Coque E, Patin F, Vourc'h P, Marouillat S, et al. Wildtype motoneurons, ALS-Linked SOD1 mutation and glutamate profoundly modify astrocyte metabolism and lactate shuttling. Glia. 2017;65:592–605.
